# Searching for the causal effects of body mass index in over 300 000 participants in UK Biobank, using Mendelian randomization

**DOI:** 10.1371/journal.pgen.1007951

**Published:** 2019-02-01

**Authors:** Louise A. C. Millard, Neil M. Davies, Kate Tilling, Tom R. Gaunt, George Davey Smith

**Affiliations:** 1 MRC Integrative Epidemiology Unit at the University of Bristol, Bristol, United Kingdom; 2 Department of Population Health Sciences, Bristol Medical School, University of Bristol, United Kingdom; 3 Intelligent Systems Laboratory, Department of Computer Science, University of Bristol, Bristol, United Kingdom; Institute for Molecular Medicine Finland (FIMM), FINLAND

## Abstract

Mendelian randomization (MR) has been used to estimate the causal effect of body mass index (BMI) on particular traits thought to be affected by BMI. However, BMI may also be a modifiable, causal risk factor for outcomes where there is no prior reason to suggest that a causal effect exists. We performed a MR phenome-wide association study (MR-pheWAS) to search for the causal effects of BMI in UK Biobank (n = 334 968), using the PHESANT open-source phenome scan tool. A subset of identified associations were followed up with a formal two-stage instrumental variable analysis in UK Biobank, to estimate the causal effect of BMI on these phenotypes. Of the 22 922 tests performed, our MR-pheWAS identified 587 associations below a stringent P value threshold corresponding to a 5% estimated false discovery rate. These included many previously identified causal effects, for instance, an adverse effect of higher BMI on risk of diabetes and hypertension. We also identified several novel effects, including protective effects of higher BMI on a set of psychosocial traits, identified initially in our preliminary MR-pheWAS in circa 115,000 UK Biobank participants and replicated in a different subset of circa 223,000 UK Biobank participants. Our comprehensive MR-pheWAS identified potential causal effects of BMI on a large and diverse set of phenotypes. This included both previously identified causal effects, and novel effects such as a protective effect of higher BMI on feelings of nervousness.

## Author summary

Mendelian randomization uses genetic variants associated with an exposure to investigate causality. For example, there are several genetic variants that have been robustly associated with body mass index, and these can be used to test whether body mass index causally affects other traits. Usually, Mendelian randomization studies focus on particular outcomes, for instance, estimating the causal effect of body mass index on blood pressure. However, a trait (such as body mass index) may be a modifiable, causal risk factor for outcomes where there is no prior reason to suggest that a causal effect exists. We recently published a novel tool called PHESANT, that allows comprehensive phenome scans to be performed, allowing researchers to search for the causal effects of a trait of interest across over 22,000 traits in UK Biobank. In this work we used PHESANT to search for the causal effects of body mass index. We identified previously established effects, for example, adverse effects of higher body mass index on risk of diabetes and hypertension. We also identified novel effects, such as a protective effect of higher body mass index on feelings of nervousness, and these need replicating in an independent sample and using other study designs.

## Introduction

Body mass index (BMI), taken to be a general indicator of adiposity, has been associated with many traits and diseases [1–3]. These observational associations may be due to a causal effect of adiposity (as reflected in BMI), a causal effect of the phenotype on BMI, confounding, or a combination of these. Mendelian randomization (MR) estimates the causal effect of an exposure on an outcome using genetic variants as an instrumental variable for the exposure [4,5]. To date, MR has been used to assess whether BMI causally affects a vast array of phenotypes [6–21]. These results suggest that higher BMI leads to an earlier age at menarche [15], a reduction in physical activity [6], an increased risk of coronary heart disease [22], asthma [13] and cancer [23], a lower risk of Parkinson’s disease [9], and changes in metabolite concentrations [7].

Hypothesis-free searching is an established method to identify novel associations, such as genetic variants associated with a particular phenotype in genome-wide association studies (GWAS) [24]. In contrast to hypothesis-driven analyses, a hypothesis-free analysis can identify novel associations where there is no prior expectation that an association might exist, and should help to avoid publication bias as all results are published, not just the most “statistically significant”. Phenome scans are a class of hypothesis-free scan that test the association of a variable of interest with a potentially large array of phenotypes—the “phenome” [25]. Phenome scans commonly seek to identify phenotypes associated with a genetic variant (phenome-wide association studies—pheWAS) or an observed phenotype (environment-wide association studies—EnWAS). A third type of phenome scan, MR-pheWAS, uses MR to search for the causal effects of a particular exposure [26–28].

To date, only a small number of phenome-wide scans of BMI have been published. We recently published a MR-PheWAS that searched for the causal effects of BMI in circa 8000 participants in the Avon Longitudinal Study of Parents and Children (ALSPAC) cohort, across 172 outcomes [26]. This study confirmed several known associations such as with leptin levels and blood pressure, and identified potentially novel associations, such as with a self-worth score. Cronin et al. performed a pheWAS of *FTO* genetic variants within electronic health record phenotypes, in circa 25 000 participants, and identified novel associations [29]. For instance, a genetic predisposition to a higher BMI was associated with a higher risk of fibrocystic breast disease and non-alcoholic liver disease [29].

UK Biobank is a prospective cohort of circa 500 000 participants [30], and several hypothesis-driven MR analyses of BMI have been performed in this study, to date [12,13,31,32]. The large sample size offsets the multiple testing burden of hypothesis-free analyses, providing an opportunity to search for causal effects with MR-pheWAS. Recently, we published an open-source tool for performing phenome scans (including MR-pheWAS) in UK Biobank—the PHEnome Scan ANalysis Tool (PHESANT) [27]. PHESANT enables comprehensive phenome scans to be performed across a large and diverse set of phenotypes—all continuous, integer and categorical fields in UK Biobank—where previously researchers would restrict their analysis to a homogeneous subset of phenotypes that could be processed in a consistent fashion [27].

In this study we search for the causal effects of adiposity, using the PHESANT tool in UK Biobank. We use BMI as a surrogate measure of adiposity. In our initial presentation of PHESANT [27] we analyzed the non-random sample of circa 115 000 participants with genetic data that was available in UK Biobank at that time. We found that participants with a genetic propensity to a higher BMI were less likely to perceive themselves as a nervous person or to call themselves tense or ‘highly strung’ [27]. In the current paper, we search for the causal effects of BMI in circa 330 000 participants in UK Biobank satisfying our inclusion criteria. BMI is a well-studied phenotype, hence this study serves as a model for future MR-pheWAS that may investigate phenotypes with much weaker priors regarding their causal effects.

## Results

We checked the strength of the association between the BMI allele score and BMI, and found a standard deviation (SD) increase in BMI allele score was associated with a 0.64 kg/m^2^ increase in BMI (95% confidence interval (CI): 0.62, 0.65, F statistic = 6114.52). Estimates of the effect of each SNP on BMI are given in Table A in S1 Text.

### Results of MR-pheWAS analysis

**Fig 1:**
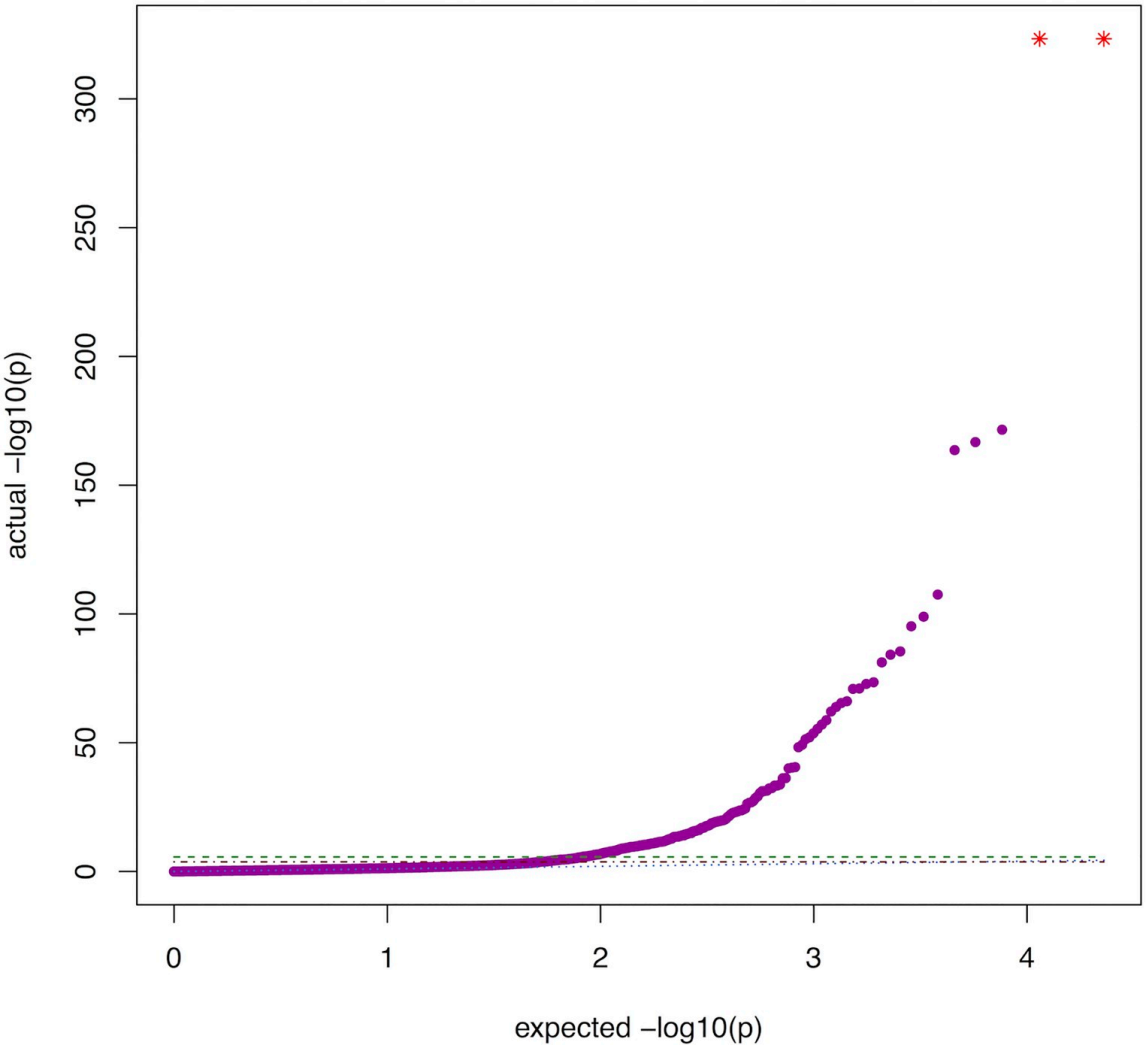
QQ plot of 22,922 MR-pheWAS results. Green dashed line: Bonferroni corrected threshold (p = (0.05/22922 = 2.18x10^-6^). Red dash-dotted line: FDR threshold (p = 0.05 × 587/22922 = 1.28x10^−3^). Blue dotted line: actual = expected. Purple points: results of tests performed in MR-pheWAS. Red stars: results with P values < 2.23x10^-308^ (the smallest number allowed in R, specified in .Machine$double.xmin).

The results of our MR-pheWAS include 22 922 tests ranked by P value, given in S1 File. The number of fields reaching each stage of the PHESANT automated pipeline are shown in Fig B in S1 Text. A QQ plot is given in Fig 1, and the PHESANT-viz visualization can be found at [http://www.datamining.org.uk/PHESANT/results-bmi-21753.html]. We identified 587 results at a false discovery rate (FDR) of 5% (using a P value threshold of 0.05 × 587/22922 = 1.28x10^−3^), given in Table D in S1 Text, and of these, 278 results had a P value lower than a stringent Bonferroni corrected threshold of 2.18x10^-6^ (0.05/22922). We estimated that there are 19,602 effectively independent phenotypes represented by the 22,879 continuous, ordered and binary outcomes using spectral decomposition. Accounting also for the 43 unordered phenotypes (for which correlations could not be generated) our correlation-based threshold was calculated as *0*.*05/(19602+43) = 2*.*55x10*^*-6*^. We found 281 results with a P value lower than this threshold.

**Fig 2:**
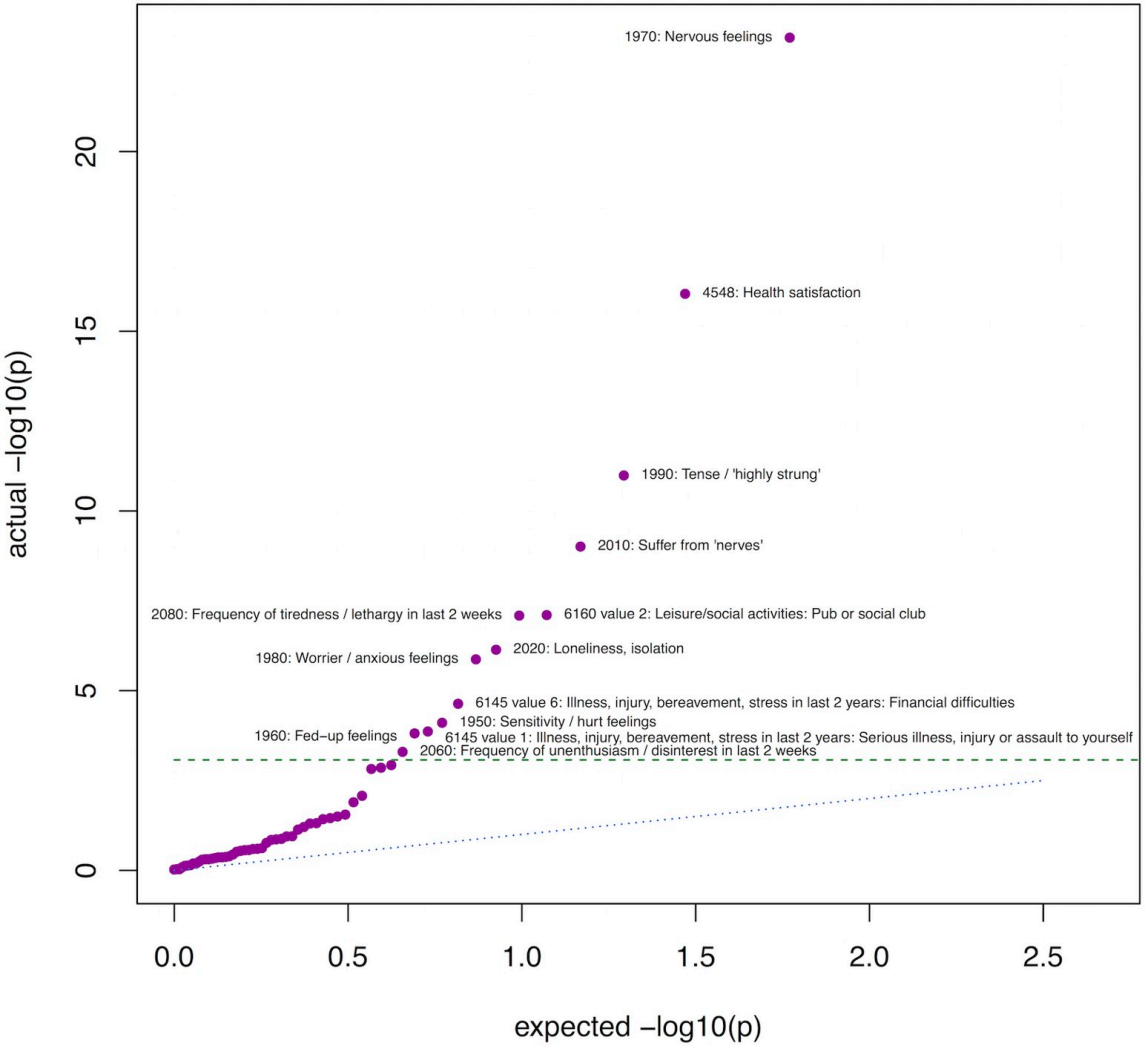
QQ plot of results in UK Biobank field category ‘psychosocial factors’ (ID 100059). Green dashed line: Bonferroni corrected threshold calculated for psychosocial traits only (0.05/59 = 8.47 x 10^−4^). Blue dotted line: actual = expected. Purple points: results of tests performed in MR-pheWAS.

Our MR-pheWAS identified several known effects of BMI. For example, a genetic predisposition to a higher BMI was associated with an increased risk of diabetes [11] (field ID (FID) = 2443), hypertension [33] (FID = 20002 value 1065, and FID = 4079), a higher bone mineral density [14] (FID = 3148) and an earlier age of puberty in both sexes [15] (FIDs = {2714, 2375, 2385}). For instance, PHESANT estimated that participants with a higher genetic risk score were on average more likely to report being diagnosed with diabetes (odds ratio [OR] per 1SD higher BMI genetic risk score of 1.147 [95% CI: 1.128, 1.165]) and more likely to report being diagnosed with hypertension (OR per 1SD higher BMI genetic risk score of 1.077 [95% CI: 1.068, 1.085]). Fig 2 shows that, when restricting to psychosocial traits, a subset is associated more strongly than would be expected by chance including several nervousness / anxiety traits.

#### PHESANT sensitivity analysis

Our tests of association of assessment centre with the BMI genetic score found that, while adjusting for the genetic principal components attenuated the association, an association remained (likelihood ratio test P values of 4.45x10^-26^, 4.32x10^-10^ and 9.57x10^-6^ when adjusting for age and sex only, and additionally the first 10 and first 40 genetic principal components, respectively). The results of our sensitivity MR-pheWAS (additionally adjusting for assessment centre and genetic batch) are given in S2 File. Comparison of the results of our main MR-pheWAS analysis (adjusting for age, sex and the first 10 genetic principal components) with our sensitivity analysis (additionally adjusting for assessment centre and genetic batch), is shown in Fig C in S1 Text. Large differences between these results occurred for binary outcomes with small numbers in a certain category (see examples in Table H in S1 Text).

### Detailed follow-up of potentially novel results

**Fig 3:**
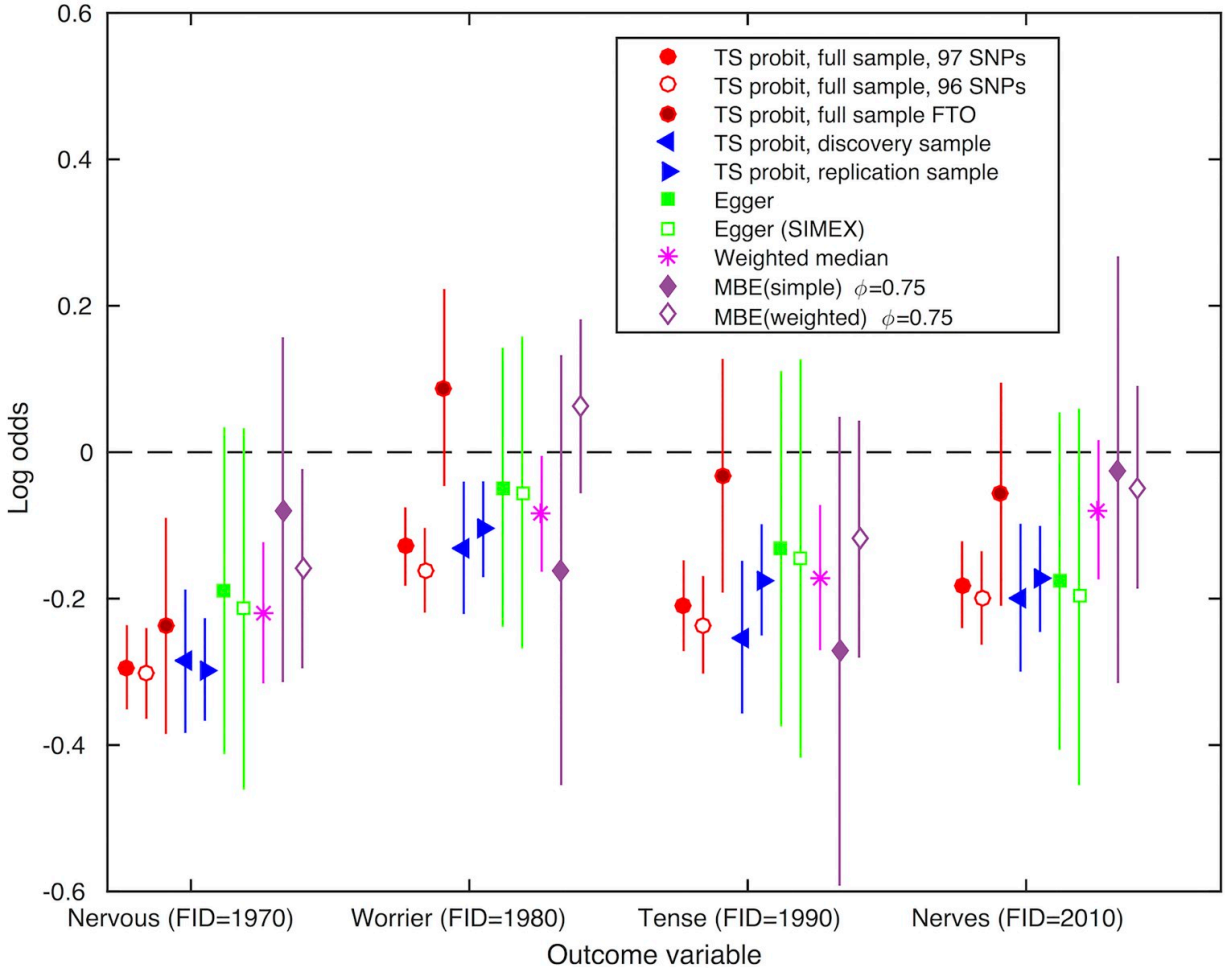
Results of follow-up analysis of the associations between nervousness / worrying traits (outcomes) and genetically predicted BMI (exposure). BMI: body mass index; FID: field identifier; SD: standard deviation; IV: instrumental variable; TS: two stage; SIMEX: simulation extrapolation; MBE: mode-based estimate. Estimates are in terms of the log odds of outcome variable for a 1SD higher BMI. TS probit analyses are adjusted for age, sex and first 10 genetic principal components (see Table F in S1 Text for results of sensitivity analysis, adjusting for age, sex and first 40 genetic principal components). TS Probit estimates of the odds ratio of outcome for a SD higher BMI are calculated by taking the exponent of 1.6 times the probit estimate [34]. TS probit, full sample, 97 SNPs: Estimates using two-stage IV probit regression using our 97 SNP score as an instrument for BMI, and our full sample (N = 334,968). TS probit, full sample, 96 SNPs: Estimates using two-stage IV probit regression using our 96 SNP score (excluding *FTO* SNP) as an instrument for BMI, and our full sample (N = 334,968). TS probit, full sample, FTO: Estimates using two-stage IV probit regression using *FTO* SNP as an instrument for BMI, and our full sample (N = 334,968). TS probit, discovery sample: Estimates using two-stage IV probit regression using our 97 SNP score as an instrument for BMI and our discovery sample. TS probit, replication sample: Estimates using two-stage IV probit regression using our 97 SNP score as an instrument for BMI and our replication sample. Discovery results are estimated on the UK Biobank sample used in the PHESANT application note usage example [27]. Sample size for discovery samples: Field 1970: 111,746; Field 1980: 111,673; Field 1990: 111,161; Field 2010: 110,451. Sample size for replication samples: Field 1970: 216,311; Field 1980: 216,301; Field 1990: 215,435; Field 2010: 214,152. F-statistics for association of 97 SNP, 96 SNP (excluding FTO) and FTO genetic instruments with BMI are 6114.52, 5200.41 and 901.91, respectively. See Table E in S1 Text for full results.

Our hypothesis-free scan found that a genetic predisposition to a higher BMI was associated with a decreased reporting of being a nervous person (FID = 1970, p = 6.79x10^-24^), being tense or ‘highly strung’ (FID = 1990, p = 1.02x10^-11^), suffering from ‘nerves’ (FID = 2010, p = 9.73x10^-10^), or being a worrier (FID = 1980, p = 1.34x10^-6^). Fig 3 and Table E in S1 Text present the causal effects of BMI on these nervousness traits, estimated using two-stage instrumental variable analyses. A higher genetically predicted BMI was associated with lower risk of reporting as a ‘nervous’ person (OR per 1kg/m^2^ higher BMI of 0.940 [95% CI: 0.929, 0.951]). A higher genetically predicted BMI was associated with a lower ‘worry’ score (OR per 1kg/m^2^ higher BMI of 0.957 [95% CI: 0.947, 0.966]). A higher BMI was observationally associated with a lower risk of self-reporting as a ‘nervous’ person (OR per 1kg/m^2^ higher BMI of 0.964 [95% CI: 0.962, 0.966]). Adjusting for age, sex and the first 40 genetic principal components did not meaningfully affect the results (see Table F in S1 Text). Furthermore, causal estimates using the discovery subsample of UK Biobank (used in our initial presentation of PHESANT [27]) and replication subsample (comprising additional participants used in this study) were consistent for these four nervousness/anxiety phenotypes. Observationally, a higher BMI was associated with a lower risk of reporting as a ‘nervous’ person, a worrier, tense/highly strung and suffering from nerves (see Table E in S1 Text). Those reporting yes for each nervousness phenotype on average were younger, live in an area with greater deprivation and left full time education earlier, and more likely to have ever been depressed a whole week (see Table I in S1 Text). Directions of associations with smoking status and sex differed across our four nervousness phenotypes. For instance, participants who reported ‘suffering from nerves’ were more likely to be male compared to those who did not report this, while participants who reported being a nervous person, being a worrier or being tense/highly strung were more likely to be female than those who did not.

As a sensitivity analysis, we estimated causal effects of BMI using two-sample MR methods. Overall, these results are broadly consistent with the results of our main one sample analyses, although the confidence intervals of most of these two-sample estimates include the null. MR-Egger regression and simulation extrapolation (SIMEX) plots are shown in Fig D and Fig E in S1 Text, respectively. We found little evidence of directional pleiotropy, as all confidence intervals for intercept estimates of MR-Egger included zero. We found evidence that the MR-Egger ‘NO Measurement Error’ (NOME) assumption—that SNP-exposure associations are measured without error—was not fully satisfied ($I_{GX}^{2}$ statistic = 0.892). However, estimates using MR-Egger with SIMEX—a correction for violation of the NOME assumption—were highly consistent to the results of MR-Egger without SIMEX.

The smoothed empirical densities of the SNP effect estimates using mode-based estimator (MBE) smoothing parameter ϕ values of 0.5, 0.75 and 1 are shown in Fig F and Fig G in S1 Text. We choose to use ϕ = 0.75 as this provided sufficient smoothing while still allowing multiple peaks to be captured. The effect estimates across IV methods—IV probit regression, and the two-sample approaches; MR-Egger, weighted median and MBE—were broadly consistent. We did, however, see a difference between the estimates of the IV probit regression and weighted MBE (*ϕ = 0*.*75*) for reporting ‘being a worrier’ (FID = 1980), with ORs per 1SD higher genetically predicted BMI of 0.879 [95% CI: 0.834, 0.926] and 1.065 [95% CI: 0.947, 1.197], respectively. The causal estimates using the 96 SNP score (F statistic = 5200.41) versus a BMI-associated SNP in *FTO* (“*FTO* SNP”; F statistic = 901.91) were consistent for the ‘being a nervous person’ (FID = 1970; heterogeneity P value = 0.421) and ‘suffering from “nerves”‘ (FID = 2010; heterogeneity P value = 0.089) outcomes, but not for reporting ‘being a worrier’ (FID = 1980; heterogeneity P value = 0.001) and ‘being tense or “highly strung” (FID = 1990; heterogeneity P value = 0.020).

The results of these four nervousness/anxiety traits do not provide a form of replication for each other because these traits are highly correlated (see Table G in S1 Text; all accuracy between 57.59% and 83.49%) and so are more likely to agree compared to tests using independent traits or a replication on an independent dataset.

We searched for other nervousness related traits by searching for the terms: “worr”, “nerv”, “tens”, “anxi”, in our results listing, to determine if other related traits were tested and an association not identified, to assess the strength of the evidence when considering results for all similar phenotypes. This identified three self-reported phenotypes describing: 1) whether the participant has seen a psychiatrist for nerves, anxiety, tension or depression, (FID = 2100; estimated OR per 1kg/m^2^ higher genetically predicted BMI of 1.009 [95% CI: 0.995, 1.024]), 2) whether the participant has seen doctor (GP) for nerves, anxiety, tension or depression" (FID = 2090; estimated OR per 1kg/m^2^ higher genetically predicted BMI of 1.006 [95% CI: 0.994, 1.017]), and 3) the frequency of tenseness / restlessness in last 2 weeks (FID = 2070; not at all versus several days or more; estimated OR per 1kg/m^2^ higher genetically predicted BMI of 0.985 [95% CI: 0.974, 0.997]).

## Discussion

In this study we used the PHESANT phenome scan tool to search for the causal effects of BMI—a MR-pheWAS analysis—in a sample of circa 330 000 participants in UK Biobank, across over twenty thousand diverse phenotypes. This systematic approach helps to avoid biases associated with hypothesis-driven analyses, where a researcher might try several tests of association until a desired result is found, and should help to avoid publication bias as all results are published, not just the most “statistically significant” [34].

Our results include associations consistent with previous MR studies, such as adverse effects of higher BMI on risk of diabetes [11] and hypertension [33], a higher bone mineral density [14] and an earlier age of puberty in both sexes [15]. Consistent with our preliminary results [27], we identified an association with a set of nervousness phenotypes, where a genetic predisposition to a higher BMI was associated with a person being less likely to call themselves a nervous person, tense or highly strung, a worrier, or to report they suffer from ‘nerves’ (consistent with results of genetic correlations in UK Biobank reported previously [35]). We followed up this analysis to estimate causal effects and found, for example, an OR of 0.940 [95% CI: 0.929, 0.951] per 1 kg/m^2^ higher genetically predicted BMI, for self-reporting as a nervous person. The causal estimates were more extreme than the observational estimates for three of these outcomes. Furthermore, causal estimates using the discovery and replication subsamples of UK Biobank were consistent for these four nervousness/anxiety phenotypes.

The four nervousness phenotypes we identified are part of the 12-item Neuroticism scale of the Eysenck Personality Questionnaire-Revised (EPQ-R) [35,36], and as we and others have shown, are highly correlated [35]. Neuroticism is one of the “Big Five” personality traits [37], and these are hierarchical—questionnaire items define facets of neuroticism, which themselves define the neuroticism personality trait as a whole [38]. Previous studies in UK Biobank have shown that these particular items form an anxiety facet of neuroticism [35,38]. For instance, Nagel et al. identified two genetically homogeneous clusters (where the within cluster genetic correlation was much higher than the between cluster genetic correlation) within the 12 neuroticism items, one of which denoted ‘worry’ and comprised of the 4 ‘nervousness’ items we identified [35]. We found that a genetic predisposition to a higher BMI was associated with a lower ‘worry’ score.

We searched our results for other nervousness traits to determine the strength of the evidence in the context of the results of related phenotypes, which, while not a form of replication, provides an unbiased view of the evidence using the UK Biobank cohort. We identified two phenotypes detailing treatment by a psychiatrist or a doctor, respectively, for nerves, anxiety, tension or depression, but we did not find evidence of a causal effect of BMI on these phenotypes. A third result, the frequency of tenseness / restlessness in the last 2 weeks, was weakly associated in a direction consistent with the other nervousness associations we identified, although it did not pass the P value threshold corresponding to an estimated FDR of 5%. The ‘null’ associations of our BMI genetic risk score with risk of seeing a doctor or psychiatrist for nerves, anxiety, tension or depression may be because, compared to our self-reported nervousness items, these phenotypes pertain to more severe manifestations where treatment has been instigated. Furthermore, worry and depressed affect have been shown to be distinct clusters of neuroticism, having negative and positive genetic correlations with BMI, respectively [35], such that combining treatment of worry and depression into a single phenotype may mean the effect of BMI on the former is masked by the effect on the latter (and vice-versa).

Several previous observational studies have reported the association of anxiety and BMI [39–45]. The prevalence of anxiety has been shown to be higher in obese compared with non-obese people [39–44] and higher in both underweight and overweight/obese people compared with those of normal weight [45]. The few instrumental variable studies estimating the causal effect of BMI on anxiety that have been performed to date (one specifically looking at phobic anxiety [46] and the other using an anxiety measure defined using the Hospital Anxiety and Depression Scale [HADS] ^50^), did not find evidence of a causal effect, although this may be due to insufficient statistical power [46,47]. Some previous MR studies using a BMI genetic risk score provided evidence that an increase in BMI adversely affects risk of depression symptoms [48] and major depressive disorder [49] while others found little evidence [50,51]. A recent study in UK Biobank that also used a BMI genetic risk score identified an adverse effect of higher BMI on wellbeing (defined as a combination of life satisfaction and happiness), but this was driven by an effect on the `satisfaction with health’ component of wellbeing, with little evidence of an effect on happiness [52]. Other studies have used a SNP in the *FTO* gene as a genetic instrument for BMI and identified potential effects of higher BMI on lower psychological distress [53] and a combined depression anxiety symptoms score [54]. However, FTO may be an invalid instrument for BMI for mental health outcomes due to horizontal pleiotropy [50,51], and this is consistent with the differences we observed between causal estimates using FTO and 96 SNP genetic risk scores for two of our nervousness outcomes. It has been shown that the mechanism through which BMI-associated genetic variants affect risk of obesity may involve regions of the brain such as the basal ganglia [55], which are plausibly involved in emotional processes [56]. While instrumental variable studies assessing potential mechanisms are lacking, one recent MR study, in addition to replicating the adverse effect of higher BMI on depression in UK Biobank, explored the pathways through which this effect may be acting [57]. They found evidence of a causal effect of BMI on depression via non-metabolic pathways. An effect of BMI on our identified worry/nervousness phenotypes may act through physiological or psychological pathways, and further work is needed to investigate this.

The associations we identified with self-reported nervousness should be further investigated and replicated in an independent sample, although at present we are not aware of a study with sufficient sample size and data available to do this. These associations may reflect a true causal effect of BMI. Alternative explanations for these results include chance, or because some variants in the BMI genetic score have horizontal pleiotropic effects and are thus invalid instruments for BMI. We ‘discovered’ and ‘replicated’ our identified associations in separate subsamples of UK Biobank suggesting our results are not due to chance. We assessed the possibility that our instrument is invalid by comparing the estimates of two independent genetic instruments (i.e. *FTO* SNP versus the remaining genetic variants), and using alternative approaches that estimate the causal effects under differing assumptions of instrument validity—MR-Egger, weighted median and MBE. Our two-sample results were broadly consistent with the results of our main (one sample) analyses, although these approaches (apart from IVW) lacked power and confidence intervals included the null. While we found little evidence of directional pleiotropy, we did find evidence of non-directional pleiotropy for the ‘being a worrier’ and being tense/“highly strung” outcomes, indicated by different estimates using the 96 SNP score versus *FTO* SNP, and also different estimates using IV probit regression compared to the weighted MBE for the former only. Instrument validity is likely to be consistent in our discovery and replication subsamples of UK Biobank as they are likely to have similar confounding structures, but may be different in other populations such that replicating in other large cohorts may be informative. Furthermore, studies assessing the effect of BMI on nervousness using other study designs (e.g. randomized controlled trials of a weight loss intervention or within-sibship studies) with different sources of potential bias would be beneficial to strengthen evidence through triangulation [58].

UK Biobank is a highly-selected sample of the UK population, having a response rate of 5.5% [59], that is not representative of the UK general population [60]. For example, UK Biobank participants have, on average, a lower BMI, and fewer self-reported health conditions, compared with the general population [60]. Hence, our estimates may be biased if selection into the sample is affected by BMI [61]. Also, if selection is additionally dependent on a given outcome (e.g. self-reported nervousness), associations may be biased by a particular form of collider bias—selection induced collider bias [62]. In general, collider bias may occur when two variables (A and B) independently affect a third variable (C) and variable C is conditioned upon in analyses. Selection induced collider bias may occur when variable C represents whether a person is selected into the sample, i.e. variables A and B both independently affect participation in the study. Hence, estimates of association between two phenotypes—such as our BMI genetic score and a given outcome in our study—can be biased, if inclusion in the study is affected by both phenotypes.

We found that our BMI genetic score was associated with assessment centre, even after adjustment for the first 40 principal components. This may indicate that the genetic principal components are not fully accounting for genetic population differences. However, this may also be due to selection induced collider bias, because both BMI and location are related to selection into the sample [60]. If both BMI and assessment centre affect participation in the study, then an association may be induced between the BMI genetic score and assessment centre. For example, the South West region had the highest participation rate of the regions sampled, such that living in this region is associated with a higher chance of participating compared to the other regions [60]. Since BMI is negatively associated with participation in UK Biobank, we would expect (under most realistic assumptions about the association between BMI, location and participation in UK Biobank) to see a positive association between the BMI genetic risk score, and participating in the South West region compared to other regions if collider bias is the cause, and (using attendance at the Bristol assessment centre as a proxy for region of residence) this is indeed what we see. Furthermore, the West Scotland region has the lowest participation rate, and attending the Glasgow assessment centre compared to other centres is associated with a lower BMI genetic risk score, which is expected if this relationship is due to selection induced collider bias.

We now discuss some further limitations of this work. PHESANT uses a rule-based method to automatically determine how to test each outcome, and it is possible that this may deal with some variables inappropriately. Also, PHESANT tests the linear association of the genetic score with the set of outcomes, and it is possible that non-linear associations may exist. For instance, it is possible that BMI has a non-linear effect on nervousness/anxiety (for example low and high BMI may cause higher levels of anxiety compared with those in the normal range) and in follow-up analyses this should be investigated. Ranking the associations means that we should expect the true strength of the associations to be less than we reported due to the winner’s curse. We used stringent P value thresholds, which, although reducing the type I error rate, is likely to also increase the type II error rate. We used BMI as a surrogate measure for adiposity, but the effect of adiposity estimated using observational data is not the true effect of an intervention that modifies BMI, because this will depend on the particular intervention used. The causal effects we have estimated may be the result of changes of other aspects of BMI in addition to or instead of changes in adiposity (e.g. changes in lean mass versus fat mass) [63]. Similarly, our BMI genetic score is an instrument for life-long BMI, hence we cannot say that it is BMI at a specific age (e.g. the age of the UK Biobank cohort) that affects an outcome. However, searching for potential causal effects using MR is useful to identify outcomes that may be modified through interventions on modifiable determinants of adiposity (e.g. diet or physical activity) [63].

PHESANT is the first tool to perform comprehensive phenome scans, where previously the set of outcomes tested would be restricted to a homogeneous subset [27]. In this study, we have presented the first comprehensive phenome scan, using the PHESANT tool to search for the causal effects of BMI. While our previous MR-pheWAS of BMI in the Avon Longitudinal Study of Parents and Children (ALSPAC) [26] searched across 172 phenotypes measured predominantly in childhood, this current study searched across over 22,000 potential outcomes recorded in later adulthood. The current study also builds on an initial BMI MR-pheWAS we conducted in UK Biobank which illustrated how the PHESANT tool can be used as part of a “Software Application Profile” [27]. While our “Software Application Profile” MR-pheWAS searched across circa 10,000 phenotypes in a non-random subsample of UK Biobank, our formal MR-pheWAS study presented here searched across more than twice the number of phenotypes and using circa three times as many participants. We confirmed several established effects of BMI, and replicated findings such as a potential causal effect of BMI on feelings of nervousness (initially identified in our “Software Application Profile” MR-pheWAS [27]). We performed comprehensive follow-up analyses to investigate violations of instrumental variable assumptions. Our previous ALSPAC MR-pheWAS also identified a novel potential effect of BMI on a psychosocial outcome; a genetic predisposition to a higher BMI was associated with higher feelings of self-worth. In ALSPAC, only a very weak negative correlation (Pearson’s rho = -0.07 [26]) is seen between feelings of self-worth and feelings of worry, suggesting that our results for worry/nervousness and self-worth pertain to different aspects of personality. Our MR-pheWAS in ALSPAC and UK Biobank demonstrate how multiple hypothesis-free studies in different populations can build a picture of the landscape of causal effects across both the phenome and the life course.

This work demonstrates how MR-pheWAS can be applied in UK Biobank and can serve as a model for future studies. There is much potential to use MR-pheWAS to search for the causal effects of phenotypes where, compared to BMI, we have much weaker priors regarding their causal effects. Phenome-wide scans are a hypothesis generation approach and hence identified associations should be followed-up in an independent sample and using other study designs with different assumptions and potential biases.

## Materials and methods

### Study population

UK Biobank sampled 503 325 men and women in the UK aged between 37–73 years (99.5% were between 40 and 69 years) [64]. This cohort includes a large and diverse range of data from blood, urine and saliva samples and health and lifestyle questionnaires [30]. UK Biobank has received ethics approval from the UK National Health Service’s National Research Ethics Service (ref 11/NW/0382).

**Fig 4:**
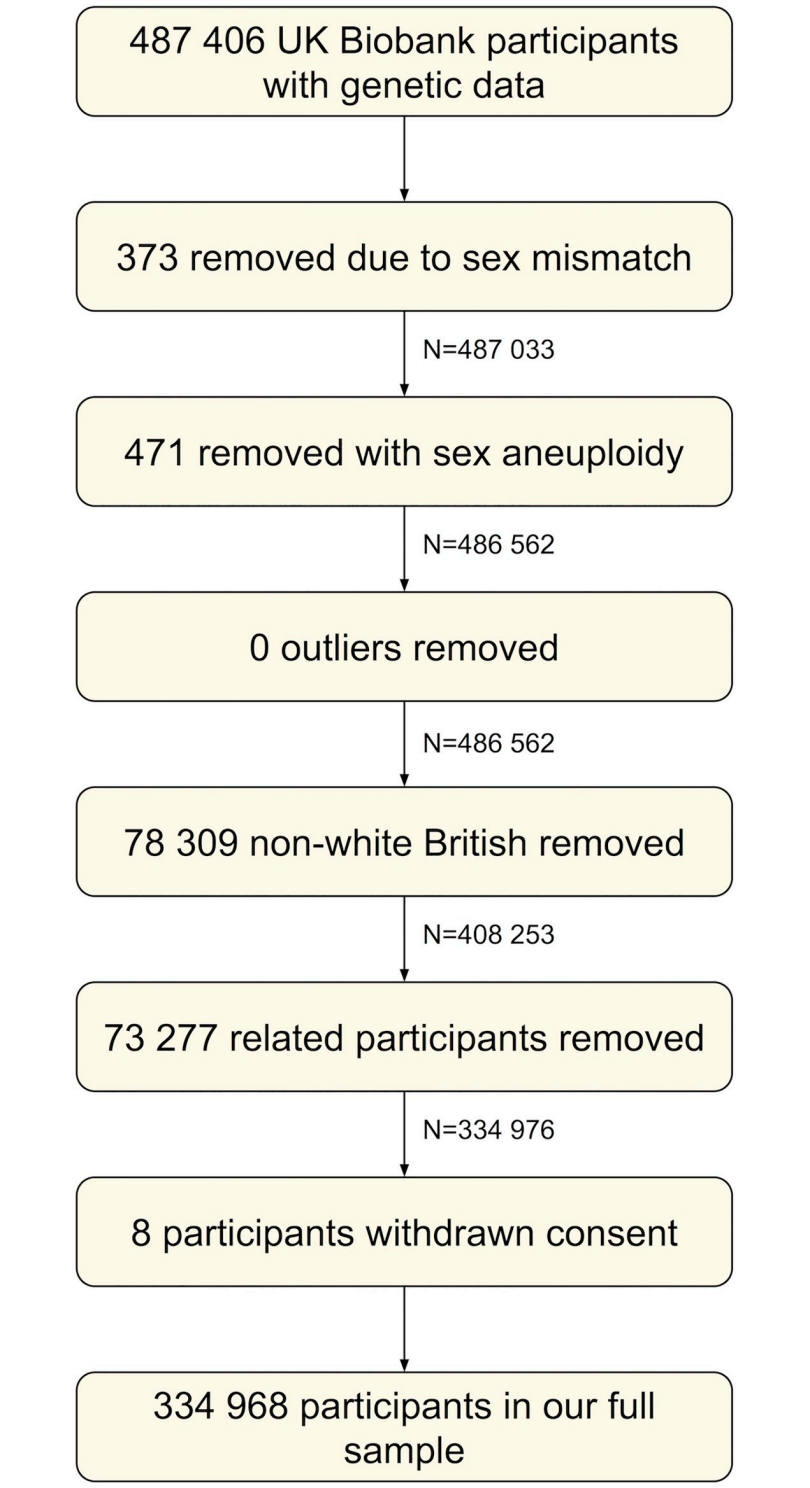
Participant flow diagram.

Of the 487 406 participants with genetic data, we removed 373 with genetic sex different to reported sex, and 471 with sex chromosome aneuploidy (identified as putatively carrying sex chromosome configurations that are not either XX or XY). We found no outliers in heterozygosity and missing rates, which would indicate poor quality of the genotypes. We removed 78 309 participants not of white British ancestry [65]. We removed 73 277 participants who were identified as being related, having a kinship coefficient denoting a third degree (or closer) relatedness [65]. We removed 8 individuals with withdrawn consent, giving a sample of 334 968 participants (of which 86 134 were in our initial PHESANT sample and 26 119 were related to our initial PHESANT sample—see Venn diagram in Fig A in S1 Text [27]). A participant flow diagram is given in Fig 4. All analyses except where stated otherwise use this ‘full’ sample of 334,968 participants.

### BMI allele score

We created an allele score from 97 genetic variants previously found to be associated with BMI, in a recent GWAS meta-analysis by the GIANT consortium [66]. The score was calculated as a sum of the number of BMI-increasing alleles, weighted by the effect size as reported in the GIANT GWAS (reported as a SD change of BMI per dosage increase; see Table A in S1 Text) [66], such that a higher allele score corresponds to a higher BMI, and was standardized to have a mean of zero and SD of 1.

### Outcomes

The Biobank data showcase allows researchers to identify variables based on the field type (http://biobank.ctsu.ox.ac.uk/showcase/list.cgi). At the time of data download (dataset 21753 in application 16729) there were 2761 fields of the following types: integer, continuous, categorical (single) and categorical (multiple). These fields pertain to a diverse range of phenotypes from blood and saliva assays, clinical assessments (anthropometry, cognitive function, hearing, arterial stiffness, hand grip strength, spirometry, ECG, carotid ultrasound, abdominal, brain and heart MRI scans, DXA scans), record linkage (cancer and death registries and hospital episodes) and health and lifestyle questionnaires (medical conditions, operations, mental health, sociodemographic factors, lifestyle, family history, psychosocial factors, dietary intake).

We excluded 74 fields a priori, given in Table B in S1 Text, for the following reasons. We removed one field denoting the assessment centre. We removed 2 fields described by UK Biobank as ‘polymorphic’, containing values with mixed data types. We removed 7 fields that, although listed in the data showcase, were not currently available. We removed 17 genetic descriptor fields, 1 sex field and 4 age fields, 17 fields describing the assessment centre environment, 4 data processing indicators, and 21 categorical (single) fields with more than one value recorded per person.

We assigned 92 outcomes that denote adiposity or some aspect of weight, fat mass or height as ‘exposure phenotypes’, identified within three field categories; “Body composition by DXA” (field category ID = 124), “Body size measures” (field category ID = 100010) and “Impedance measures” (field category ID = 100009). These are included in our MR-pheWAS analysis so that we can assess the strength of outcome associations in relation to these, while this a priori assignment means they will not contribute to the multiple testing burden (see Table C in S1 Text).

This resulted in a set of 2595 UK Biobank fields (320 integer, 1333 continuous, 830 categorical (single) and 112 categorical (multiple)), referred to hereafter as the outcome dataset (because they are tested as an outcome irrespective of whether this is biologically plausible).

### Observed BMI

Weight and height were measured at the initial UK Biobank assessment centre—weight in light clothing and unshod was measured using Tanita BC418MA body composition analyser to the nearest 100g, and height to the nearest cm using at Seca 202 device. These were used to calculate BMI (kg/m^2^).

### Covariates

We include age and sex as covariates in our models to reduce the variation in our outcomes. Age when participants attended the UK Biobank assessment centre was derived from their date of birth and the date of their assessment centre visit. Sex was self-reported during the touchscreen questionnaire (and validated using the genome-wide data). We adjust for the first 10 genetic principal components to control for confounding via population stratification. Genetic variants are set at conception, and after conception they cannot be affected by traditional confounding factors, therefore we did not adjust for any further covariates.

### Statistical methods

#### PHESANT MR-pheWAS

We test the direct association of the BMI genetic score with each of the outcome variables using the PHESANT package (version 0.17). A description of PHESANT’s automated rule-based method is given in detail elsewhere [27]. In brief, all fields are processed and analyzed separately and the decision rules start with the variable field type and use rules to categorize each variable as one of four data types: continuous, ordered categorical, unordered categorical or binary. Variables with the continuous and integer field types are usually assigned to the continuous data type, but some are assigned to ordered categorical if, for instance, there are only a few distinct values. Variables of the categorical (single) field type are assigned to either the binary, ordered categorical or unordered categorical, depending on whether the field has two distinct values, or has been specified as ordered or unordered in the PHESANT setup files. Variables of the categorical (multiple) field type are converted to a set of binary variables, one for each value in the categorical (multiple) fields. For instance, international classification of disease (ICD) codes of hospital inpatient primary and secondary diagnoses are two separate fields hence are analyzed separately by PHESANT. These ICD fields are of the categorical (multiple) field type, hence are converted to a set of binary variables denoting the prevalence of each ICD code using all data available at or after baseline, such that prevalence prior to the initial linkage to these data is combined with prospective events that occurred after the start of the study. Example fields and how they are processed by PHESANT are given in Section S1 in S1 Text, and further details are provided in our “Software Application Profile” [27].

PHESANT estimates the bivariate association of the BMI genetic score with each outcome variable. The BMI genetic score and outcome variables are the independent (exposure) and dependent (outcome) variables in the regression model, respectively. Outcome variables with continuous, binary, ordered categorical and unordered categorical data types, are tested using linear, logistic, ordered logistic, and multinomial logistic regression, respectively. Prior to testing, an inverse normal rank transform is applied to variables of the continuous data type, to ensure they are normally distributed. All analyses are adjusted for covariates as described above.

We assess the strength of the evidence of our results accounting for multiple testing using three alternative approaches. First, we calculate a stringent Bonferroni corrected P-value threshold, by dividing 0.05 by the number of tests performed, which controls the family-wise error rate (the probability of one or more type 1 errors) and assumes each test is independent. Second, we generate a P value threshold that also controls the family-wise error rate but uses an estimate of the effective number of independent phenotypes across our outcome dataset, hence is less conservative. We run PHESANT with the ‘save’ option to derive the set of 22,922 phenotypes that are tested in our phenome scan. We generate a correlation matrix for the subset of 22,879 continuous, ordered and binary phenotypes using Spearman’s rank correlations (excluding 43 unordered phenotypes for which correlations could not be calculated). We estimate the number of independent tests *T* among these 22,879 phenotypes using spectral decomposition, conducted using the PhenoSpD package [67]. We calculate the P value threshold as *0*.*05/(T+43)*, where 43 is the number of unordered categorical phenotypes for which correlations cannot be computed, and refer to this threshold as the correlation-based P value threshold. Third, we control for the expected proportion of false positive results among ‘hits’. After ranking the results by P value, we identify the largest rank position with a P value less than *P*_*threshold*_ = 0.05 × rank/n, where *n* is the total number of tests in the phenome scan. *P*_*threshold*_ is the P value threshold resulting in a FDR of 5% [68].

#### Results visualization with PHESANT-viz

We use PHESANT-viz, a D3 Javascript visualization tool included in the PHESANT package, to visualize our results as a graph, using the Biobank assigned category structure (http://biobank.ctsu.ox.ac.uk/showcase/label.cgi). This enables interpretation of the identified associations in the context of the results for related variables. For example, the estimated effects of BMI on depression are grouped with the results of other psychosocial phenotypes.

#### PHESANT sensitivity analysis

We re-run our phenome scan to assess residual confounding, additionally adjusting for both assessment centre and genetic batch to assess whether results may be biased by residual population stratification or batch effects. We include these as a sensitivity analysis only as these additional adjustments may increase bias if, for instance, genetic batch (which is known to associate with smoking in UK Biobank) or location are affected by both the genetic risk score and an outcome (collider bias [62]), or are mediators between our genetic risk score and an outcome.

#### Testing for residual population stratification

We test the extent that genetic principal components account for genetic differences across the population. We test the association of assessment centre with the BMI genetic score (as the independent and dependent variables, respectively), adjusting for:

1. Age and sex.
2. Age, sex and the first 10 genetic principal components.
3. Age, sex and the first 40 genetic principal components.

We use a likelihood ratio test to determine the strength of the association of the assessment centres collectively, with the genetic score. An association between assessment centre and the BMI genetic score, after adjusting for the genetic principal components, may indicate that the genetic principal components are not fully accounting for population stratification.

#### Follow-up analysis of identified associations

We identified associations with a related set of psychosocial traits that are part of the 12-item Neuroticism scale of the Eysenck Personality Questionnaire-Revised (EPQ-R) [35,36]. We generate a QQ-plot restricting to the psychosocial UK Biobank category only (category ID = 100059), to determine whether these results have an association stronger than expected by chance, given the results of related phenotypes. We perform a formal instrumental variable analysis for a set of binary outcomes, using two-stage IV probit regression (the Stata ivprobit command, with conditional maximum-likelihood estimation), adjusting for age, sex and the first 10 genetic principal components. We also adjust for age, sex and the first 40 genetic principal components, as a sensitivity analysis. We take the exponent of 1.6 times the estimates, to approximate the association in terms of the change of odds [69]. Our four identified nervousness phenotypes were previously identified as a homogenous set of ‘neuroticism’ items of the EPQ-R, denoting a ‘worry’ facet of the neuroticism personality trait, and used to construct a ‘worry’ score [35]. We generate this ‘worry’ score as the number of ‘yes’ responses for these four nervousness phenotypes such that the cluster scores range from zero to four, and estimate the association of this score on the BMI genetic risk score with ordered logistic regression (the Stata ologit command). We also test the observational association of BMI with each nervousness phenotype using logistic regression (Stata logistic command).

We identified the same set of psychosocial traits as in our preliminary “Software Application Profile” MR-pheWAS, which was conducted on a non-random subsample with genetic data available at that time [27]. We refer to the 114,863 sample used in our presentation of PHESANT [27] as the discovery sample (see Fig A in S1 Text). We compare estimates using this discovery sample with estimates using the additional participants used in this present study, as a replication. We remove all participants from our replication sample who were identified as being related to a participant in the discovery sample, having a kinship coefficient denoting a third degree (or closer) relatedness, giving a replication sample of 222 715 participants (see Fig A in S1 Text).

#### Follow-up sensitivity analyses

We performed sensitivity analyses to investigate whether our genetic score, and its constituent genetic variants, may be invalid instruments for BMI. First, we compared the effect estimates using two independent instrumental variables: 1) SNP rs1558902 (the SNP most strongly associated with BMI, at the *FTO* locus), and 2) the remaining 96 genetic variants.

Second, we also estimated causal effects using three alternative MR approaches—MR-Egger [70], weighted median [71] and MBE [72]. These methods estimate effects consistent with the true causal effect under more relaxed assumptions of instrument validity, compared with IV probit regression where for example, including just one genetic variant with pleiotropic effects could bias estimates. These methods are performed using two sample MR, using estimates of association of each genetic variant on BMI, and estimates of association of each genetic variant on a given outcome, respectively, estimated in different populations. We use the SNP-BMI estimates from the GIANT GWAS [66] (that did not include UK Biobank), and estimate the SNP-outcome associations on our UK Biobank sample, adjusting for age, sex and the first 10 genetic principal components.

The MR-Egger, weighted median and MBE approaches are complementary, each depending on distinct assumptions about instrument validity [73]. Estimates from MR-Egger are not biased by horizontal pleiotropy, where a genetic variant affects several traits through separate pathways, under the assumption that the association of each genetic variant with the exposure is independent of any horizontal pleiotropic effect—referred to as the InSIDE (Instrument Strength Independent of Direct Effects) assumption [70]. This approach can test for directional pleiotropy, which is when the horizontal pleiotropic effects of the genetic variants are not balanced about the null [70]. Directional pleiotropy is identified where the intercept estimate is not consistent with the null. MR-Egger assumes that the SNP-exposure associations are measured without error, known as the NOME assumption [74]. We determine the degree to which the NOME assumption is violated, using the $I_{GX}^{2}$ statistic, which is an adaption of the *I*^*2*^ statistic used in the field of meta-analysis [74]. The $I_{GX}^{2}$ statistic ranges between 0 and 1, and captures the uncertainty in the estimated SNP-exposure associations relative to the heterogeneity across the true underlying SNP-exposure associations. It is recommended that results of MR-Egger should be treated with caution when $I_{GX}^{2}<0.9$ [70]. Furthermore, we performed an MR-Egger analysis with bias adjustment using SIMEX, to estimate the causal effect when the NOME assumption is violated [74]. In brief, SIMEX works by learning models with increasing violations of the NOME assumption (i.e. increasing error in the SNP-exposure associations). The estimates from these models are then treated as a set of data points and a model is learnt across these. This ‘meta’ model is then used to extrapolate back to the estimate that would have occurred if the NOME assumption was satisfied.

The weighted median approach estimates a consistent causal effect under the assumption that less than 50% of the genetic variants are invalid instruments [71]. The simple version of MBE assumes that the mode of the smoothed empirical density function of SNP estimates is consistent with the true causal effect, even if the majority of the SNP estimates are not consistent—known as the ZEro Mode Pleiotropy Assumption (ZEMPA) [72]. We also test using a weighted version of the MBE, which instead assumes that the mode of the inverse-variance weighted empirical density function is consistent with the true causal effect. The MBE method uses a bandwidth parameter *ϕ*, which determines the amount of smoothing of the empirical density. We generate the smoothed empirical density using a range of *ϕ* values, and choose a *ϕ* value that provides an appropriate amount of smoothing (a degree of smoothing that allows multi-modal distributions to be identified while not overfitting to the estimated values). We test the simple and weighted versions of the MBE using our chosen *ϕ* value.

Analyses are performed in R version 3.2.4 ATLAS, Matlab r2015a or Stata version 14, and code is available at [https://github.com/MRCIEU/PHESANT-MR-pheWAS-BMI]. Git tag v0.5 corresponds to the version presented here.

## Supporting information

S1 Text Supplementary tables and figures. (DOCX)

S1 File Results from BMI MR-pheWAS. (CSV)

S2 File Results from sensitivity BMI MR-pheWAS (additionally adjusting for assessment centre and genetic batch). (CSV)
