# Supplementary material for: Searching for the causal effects of body mass index in over 300 000 participants in UK Biobank, using Mendelian randomization
Source: PLoS Genet. 2019 Feb 1;15(2):e1007951. doi: 10.1371/journal.pgen.1007951 (PMC6373977; doi:10.1371/journal.pgen.1007951)
Supplement: S1 Text — (DOCX) [file pgen.1007951.s001.docx]

**Searching for the causal effects of body mass index in over 300 000 participants in UK Biobank, using Mendelian randomization**

Louise A C Millard, Neil M Davies, Kate Tilling, Tom R Gaunt, George Davey Smith

SUPPLEMENTARY MATERIAL

Supplementary section S1: Examples of PHESANTs treatment of fields depending on assigned field type

**Field 40001, “underlying (primary) cause of death: ICD-10”:** This field is assigned the categorical (single) field type but is reassigned as a categorical (multiple) field in PHESANT’s variable information file. This means that PHESANT derives a binary variable for each ICD code. All participants with this code are assigned the value TRUE, all other participants are assigned the value FALSE, such that each variable denotes the prevalence of each ICD code, using all data available at or after baseline. PHESANT tests the association of each code with the BMI genetic risk score using binary logistic regression.

**Field 2443, “Diabetes diagnosed by doctor”:** This field is assigned the categorical (single) field type. PHESANT detects that this is a binary variable (‘yes’ versus ‘no’), assigns this field the binary data type and tests the association with binary logistic regression.

**Field 2178, “Overall health rating”:** This field is assigned the categorical (single) field type, and has 4 categories (poor, fair, good and excellent). PHESANT uses the manually curated variable information file to determine that the field values are ordered, and then tests the association using ordered logistic regression.

**Field 2395, “Hair/balding pattern”**: This field is assigned the categorical (single) field type and has 4 categories each corresponding to a particular balding pattern. PHESANT uses the manually curated variable information file to determine that the field values are unordered, and then tests the association using multinomial logistic regression.

**Field 30250, “Reticulocyte count”**: This field is assigned the continuous field type in UK Biobank. PHESANT uses an inverse-rank normal transformation to ensure the variable is normally distributed, and then tests the association using linear regression.

SUPPLEMENTARY TABLES

Supplementary table A: Genetic variants and weights used to construct BMI genetic score for preliminary MR-pheWAS

| SNP | Effect allele | Other allele | Effect size reported by GIANT | Effect allele frequency in our sample | Effect size in our UK Biobank sample ^1^ |
| --- | --- | --- | --- | --- | --- |
| Novel loci in Table 1 of Locke paper (1) | | | | | |
| rs657452 | A | G | 0.023 | 0.391 | 0.017 [0.012,0.022] |
| rs12286929 | G | A | 0.022 | 0.527 | 0.016 [0.011,0.021] |
| rs7903146 | C | T | 0.023 | 0.709 | 0.018 [0.012,0.023] |
| rs10132280 | C | A | 0.023 | 0.699 | 0.025 [0.020,0.030] |
| rs17094222 | C | T | 0.025 | 0.213 | 0.017 [0.011,0.023] |
| rs7599312 | G | A | 0.022 | 0.732 | 0.017 [0.012,0.023] |
| rs2365389 | C | T | 0.020 | 0.592 | 0.017 [0.012,0.022] |
| rs2820292 | C | A | 0.020 | 0.567 | 0.022 [0.017,0.026] |
| rs12885454 | C | A | 0.021 | 0.642 | 0.015 [0.010,0.020] |
| rs16851483 | T | G | 0.048 | 0.066 | 0.038 [0.028,0.047] |
| rs1167827 | G | A | 0.020 | 0.565 | 0.022 [0.017,0.027] |
| rs758747 | T | C | 0.023 | 0.278 | 0.013 [0.008,0.019] |
| rs1928295 | T | C | 0.019 | 0.570 | 0.013 [0.008,0.018] |
| rs9925964 | A | G | 0.019 | 0.642 | 0.028 [0.023,0.033] |
| rs11126666 | A | G | 0.021 | 0.256 | 0.004 [-0.002,0.009] |
| rs2650492 | A | G | 0.021 | 0.299 | 0.017 [0.012,0.022] |
| rs6804842 | G | A | 0.019 | 0.575 | 0.011 [0.006,0.016] |
| rs4740619 | T | C | 0.018 | 0.551 | 0.019 [0.014,0.023] |
| rs13191362 | A | G | 0.028 | 0.875 | 0.020 [0.013,0.027] |
| rs3736485 | A | G | 0.018 | 0.461 | 0.013 [0.008,0.018] |
| rs17001654 | G | C | 0.031 | 0.148 | 0.017 [0.010,0.024] |
| rs11191560 | C | T | 0.031 | 0.076 | 0.025 [0.016,0.034] |
| rs1528435 | T | C | 0.018 | 0.620 | 0.016 [0.011,0.021] |
| rs1000940 | G | A | 0.019 | 0.301 | 0.015 [0.009,0.020] |
| rs2033529 | G | A | 0.019 | 0.289 | 0.022 [0.017,0.028] |
| rs11583200 | C | T | 0.018 | 0.387 | 0.015 [0.010,0.020] |
| rs9400239 | C | T | 0.019 | 0.707 | 0.017 [0.012,0.023] |
| rs10733682 | A | G | 0.017 | 0.472 | 0.013 [0.008,0.018] |
| rs11688816 | G | A | 0.017 | 0.543 | 0.012 [0.007,0.017] |
| rs11057405 | G | A | 0.031 | 0.894 | 0.028 [0.020,0.035] |
| rs11727676 | T | C | 0.036 | 0.904 | 0.008 [0.000,0.017] |
| rs3849570 | A | C | 0.019 | 0.348 | 0.012 [0.007,0.017] |
| rs6477694 | C | T | 0.017 | 0.355 | 0.011 [0.006,0.016] |
| rs7899106 | G | A | 0.040 | 0.051 | 0.025 [0.014,0.036] |
| rs2176598 | T | C | 0.020 | 0.245 | 0.020 [0.015,0.026] |
| rs2245368 | C | T | 0.032 | 0.168 | 0.023 [0.017,0.030] |
| rs17724992 | A | G | 0.019 | 0.733 | 0.016 [0.011,0.021] |
| rs7243357 | T | G | 0.022 | 0.825 | 0.019 [0.012,0.025] |
| rs2033732 | C | T | 0.019 | 0.744 | 0.011 [0.005,0.016] |
| Novel loci in Table 2 of Locke paper (1) | | | | | |
| rs9641123 | C | G | 0.029 | 0.408 | 0.009 [0.004,0.014] |
| **rs7164727** | T | C | 0.019 | 0.669 | 0.016 [0.011,0.021] |
| rs492400 | C | T | 0.024 | 0.430 | 0.013 [0.008,0.018] |
| **rs2080454** | C | A | 0.017 | 0.390 | 0.010 [0.005,0.015] |
| rs7239883 | G | A | \| 0.023 \| \| --- \| | 0.378 | 0.010 [0.005,0.015] |
| **rs2836754** | C | T | 0.017 | 0.641 | 0.011 [0.006,0.016] |
| **rs9914578** | G | C | 0.020 | 0.203 | 0.010 [0.004,0.016] |
| **rs977747** | T | G | 0.017 | 0.417 | 0.018 [0.013,0.023] |
| rs9374842 | T | C | 0.023 | 0.772 | 0.012 [0.006,0.017] |
| rs4787491 | G | A | 0.022 | 0.535 | 0.021 [0.017,0.026] |
| **rs1441264** | A | G | 0.017 | 0.592 | 0.020 [0.015,0.025] |
| **rs17203016** | G | A | 0.021 | 0.202 | 0.017 [0.011,0.023] |
| rs16907751 | C | T | 0.047 | 0.905 | 0.020 [0.012,0.028] |
| **rs13201877** | G | A | 0.024 | 0.134 | 0.008 [0.001,0.015] |
| rs9540493 | A | G | 0.021 | 0.456 | 0.015 [0.010,0.019] |
| **rs1460676** | C | T | 0.021 | 0.155 | 0.013 [0.007,0.020] |
| rs6465468 | T | G | 0.025 | 0.310 | 0.005 [-0.000,0.010] |
| **rs7715256** | G | T | 0.017 | 0.426 | 0.015 [0.011,0.020] |
| rs6091540 | C | T | 0.030 | 0.702 | 0.017 [0.011,0.022] |
| rs2176040 | A | G | 0.024 | 0.352 | 0.003 [-0.002,0.008] |
| Previously known GWAS BMI loci in Extended Data Table 2 of Locke paper (1) | | | | | |
| rs1558902 | A | T | 0.082 | 0.403 | 0.074 [0.069,0.079] |
| rs6567160 | C | T | 0.056 | 0.234 | 0.051 [0.046,0.057] |
| rs13021737 | G | A | 0.06 | 0.829 | 0.054 [0.047,0.060] |
| rs10938397 | G | A | 0.04 | 0.434 | 0.030 [0.025,0.035] |
| rs543874 | G | A | 0.048 | 0.208 | 0.049 [0.043,0.055] |
| rs2207139 | G | A | 0.045 | 0.168 | 0.043 [0.037,0.049] |
| rs11030104 | A | G | 0.041 | 0.796 | 0.039 [0.034,0.045] |
| rs3101336 | C | T | 0.033 | 0.600 | 0.021 [0.017,0.026] |
| rs7138803 | A | G | 0.032 | 0.368 | 0.028 [0.024,0.033] |
| rs10182181 | G | A | 0.031 | 0.487 | 0.038 [0.033,0.043] |
| rs3888190 | A | C | 0.031 | 0.402 | 0.027 [0.022,0.032] |
| rs1516725 | C | T | 0.045 | 0.864 | 0.033 [0.026,0.040] |
| rs12446632 | G | A | 0.04 | 0.858 | 0.026 [0.019,0.033] |
| rs2287019 | C | T | 0.036 | 0.818 | 0.034 [0.027,0.040] |
| rs16951275 | T | C | 0.031 | 0.774 | 0.030 [0.024,0.035] |
| rs3817334 | T | C | 0.026 | 0.407 | 0.026 [0.021,0.030] |
| rs2112347 | T | G | 0.026 | 0.641 | 0.030 [0.025,0.034] |
| rs12566985 | G | A | 0.024 | 0.437 | 0.015 [0.010,0.019] |
| rs3810291 | A | G | 0.028 | 0.677 | 0.028 [0.023,0.033] |
| rs7141420 | T | C | 0.024 | 0.514 | 0.022 [0.017,0.026] |
| rs13078960 | G | T | 0.03 | 0.200 | 0.020 [0.014,0.026] |
| rs10968576 | G | A | 0.025 | 0.323 | 0.028 [0.023,0.033] |
| rs17024393 | C | T | 0.066 | 0.026 | 0.063 [0.048,0.078] |
| rs12429545 | A | G | 0.033 | 0.129 | 0.025 [0.018,0.033] |
| rs13107325 | T | C | 0.048 | 0.075 | 0.054 [0.045,0.063] |
| rs11165643 | T | C | 0.022 | 0.592 | 0.018 [0.013,0.023] |
| rs17405819 | T | C | 0.022 | 0.703 | 0.019 [0.014,0.024] |
| rs1016287 | T | C | 0.023 | 0.299 | 0.021 [0.016,0.026] |
| rs4256980 | G | C | 0.021 | 0.656 | 0.017 [0.012,0.022] |
| rs12401738 | A | G | 0.021 | 0.383 | 0.018 [0.014,0.023] |
| rs205262 | G | A | 0.022 | 0.268 | 0.029 [0.023,0.034] |
| rs9581854 (referred to as rs12016871 in (1)) | T | C | 0.03 | 0.181 | 0.008 [0.002,0.014] |
| rs12940622 | G | A | 0.018 | 0.559 | 0.017 [0.013,0.022] |
| rs11847697 | T | C | 0.049 | 0.034 | 0.025 [0.013,0.036] |
| rs2075650 | A | G | 0.026 | 0.853 | 0.019 [0.012,0.025] |
| rs2121279 | T | C | 0.025 | 0.126 | 0.010 [0.003,0.017] |
| rs29941 | G | A | 0.018 | 0.673 | 0.016 [0.011,0.021] |
| rs1808579 | C | T | 0.017 | 0.515 | 0.020 [0.016,0.025] |

Genetic variants and weights from Locke GWAS meta-analysis (1)

^1^ Standard deviation change in BMI for a 1 dosage increase in SNP BMI increasing allele reported in Locke GWAS meta-analysis.

**Emboldened SNPs are those identified by Locke et al.** (1) **when including individuals of non-European descent – our results here show that these SNPs are associated with BMI in our white-British subsample of UK Biobank.**

Supplementary table B: UK Biobank fields excluded from BMI MR-pheWAS

| **Number of fields** | **Field IDs** | **Reason excluded from phenome scan** |
| --- | --- | --- |
| 1 | 54 | Assessment centre |
| 2 | 87, 92 | Polymorphic fields (containing values with mixed data types) |
| 7 | 5990, 22014, 22015, 22050, 23207, 23211, 23294, 23303, 24025, 110003 | Not available at time of data download |
| 17 | 22000, 22001, 22003, 22004, 22005, 22006, 22009, 22010, 22011, 22012, 22013, 22018, 22019, 22021, 22027, 22051, 22052 | Genetic data description fields |
| 1 | 31 | Sex field |
| 4 | 34, 52, 21003, 21022 | Age fields |
| 17 | 20012, 20013, 20014, 3059, 3065, 3081, 4268, 4275, 4281, 4287, 5149, 5152, 5155, 5164, 6024, 6074, 6075 | Assessment centre environment (ACE) fields |
| 4 | 22411, 22412, 22413, 22414 | Data processing indicators |
| 21 | 4232, 4243, 4259, 5090, 5091, 5136, 5138, 5139, 5140, 5141, 5142, 5143, 5144, 5145, 5146, 5147, 5148, 6312, 401, 402, 10691 | Categorical (single) field with more than one value recorded per person. |

Supplementary table C: UK Biobank fields specified a priori as denoting adiposity or some aspect of weight

| UK Biobank field ID | | | | | | |
| --- | --- | --- | --- | --- | --- | --- |
| 21 | 21002 | 23112 | 23126 | 23254 | 23268 | 23282 |
| 48 | 23098 | 23113 | 23127 | 23255 | 23269 | 23283 |
| 49 | 23099 | 23114 | 23128 | 23256 | 23270 | 23284 |
| 50 | 23100 | 23115 | 23129 | 23257 | 23271 | 23285 |
| 3160 | 23101 | 23116 | 23130 | 23258 | 23272 | 23286 |
| 12143 | 23102 | 23117 | 23245 | 23259 | 23273 | 23287 |
| 12144 | 23104 | 23118 | 23246 | 23260 | 23274 | 23288 |
| 20015 | 23105 | 23119 | 23247 | 23261 | 23275 | 23289 |
| 20041 | 23106 | 23120 | 23248 | 23262 | 23276 |  |
| 20045 | 23107 | 23121 | 23249 | 23263 | 23277 |  |
| 20046 | 23108 | 23122 | 23250 | 23264 | 23278 |  |
| 20047 | 23109 | 23123 | 23251 | 23265 | 23279 |  |
| 20048 | 23110 | 23124 | 23252 | 23266 | 23280 |  |
| 21001 | 23111 | 23125 | 23253 | 23267 | 23281 |  |

Supplementary table D: Results from BMI MR-pheWAS below P values threshold of 1.20x10^-3^ with a false discovery rate of 5%, ranked by P value

| **Rank** | **Field ID** ^4^ | **Assoc** ^1^ | **P value ^2^** | **Regression Type** | **Field description** | **Reference, baseline or ordinal categories ^3^** |
| --- | --- | --- | --- | --- | --- | --- |
| 1 | 1687 | Increase | <2.23e-308 | ORDERED | Comparative body size at age 10 | *{thinner … plumper}* |
| 2 | 3143 | Increase | <2.23e-308 | LINEAR | Ankle spacing width | - |
| 3 | 2714 | Decrease | 2.92e-172 | ORDERED | Age when periods started (menarche) | *{3 age bands}* |
| 4 | 4100 | Increase | 1.72e-167 | LINEAR | Ankle spacing width (left) | - |
| 5 | 4119 | Increase | 2.36e-164 | LINEAR | Ankle spacing width (right) | *-* |
| 6 | 41204 value E669 | Increase | 2.94e-108 | BINARY | Diagnoses - secondary ICD10: Obesity, unspecified | No |
| 7 | 30300 | Increase | 1.22e-99 | LINEAR | High light scatter reticulocyte count | *-* |
| 8 | 30290 | Increase | 6.29e-96 | LINEAR | High light scatter reticulocyte percentage | *-* |
| 9 | 924 | Decrease | 3.23e-86 | ORDERED | Usual walking pace | *{slow … brisk}* |
| 10 | 1418 (reference=2) | - | 6.29e-85 | MULTINOMIAL | Milk type used | Semi-skimmed |
| 11 | 30280 | Increase | 6.38e-82 | LINEAR | Immature reticulocyte fraction | - |
| 12 | 6150 value 4 | Increase | 2.97e-74 | BINARY | Vascular/heart problems diagnosed by doctor: high blood pressure | No |
| 13 | 20002 value 1065 | Increase | 1.58e-73 | BINARY | Non-cancer illness code, self-reported: hypertension | No |
| 14 | 1697 | Increase | 8.02e-72 | ORDERED | Comparative height size at age 10 | *{shorter … taller}* |
| 15 | 6150 value 100 | Decrease | 1.18e-71 | BINARY | Vascular/heart problems diagnosed by doctor: “None of the above | “None of the above” option not selected |
| 16 | 30250 | Increase | 7.30e-67 | LINEAR | Reticulocyte count | *-* |
| 17 | 1538 (reference=0) | - | 3.76e-66 | MULTINOMIAL | Major dietary changes in the last 5 years | *No* |
| 18 | 30240 | Increase | 1.18e-64 | LINEAR | Reticulocyte percentage | *-* |
| 19 | 2443 | Increase | 7.65e-63 | BINARY | Diabetes diagnosed by doctor | *No* |
| 20 | 20003 value 1140884600 | Increase | 1.77e-59 | BINARY | Treatment/medication code: metformin | No |
| 21 | 1558 | Increase | 8.91e-58 | ORDERED | Alcohol intake frequency | *{daily/almost daily … never}* |
| 22 | 2178 | Increase | 4.32e-56 | ORDERED | Overall health rating | *{excellent … poor}* |
| 23 | 1498 | Increase | 2.05e-54 | ORDERED | Coffee intake (number per day) | *{3 quantity bands}* |
| 24 | 41204 value I10 | Increase | 7.06e-53 | BINARY | Diagnoses - secondary ICD10: Essential (primary) hypertension | No |
| 25 | 20002 value 1220 | Increase | 4.28e-52 | BINARY | Non-cancer illness code, self-reported: diabetes | *No* |
| 26 | 41204 value E119 | Increase | 5.83e-50 | BINARY | Diagnoses - secondary ICD10: non-insulin-dependent diabetes mellitus - without complications | *No* |
| 27 | 20536 (reference=0) | - | 5.25e-49 | MULTINOMIAL | Weight change during worst episode of depression | Stayed about the same or was on a diet |
| 28 | 2188 | Increase | 2.96e-41 | BINARY | Long-standing illness, disability or infirmity | No |
| 29 | 2375 | Decrease | 4.34e-41 | ORDERED | Relative age of first facial hair | *{Younger than average … older than average}* |
| 30 | 6177 value 2 | Increase | 8.99e-41 | BINARY | Medication for cholesterol, blood pressure or diabetes: blood pressure medication | No |
| 31 | 30070 | Increase | 5.49e-37 | LINEAR | Red blood cell (erythrocyte) distribution width | - |
| 32 | 2316 | Increase | 6.34e-37 | BINARY | Wheeze or whistling in the chest in last year | No |
| 33 | 6144 value 4 | Increase | 1.62e-34 | BINARY | Never eat sugar or foods/drinks containing sugar | No |
| 34 | 2385 | Decrease | 3.89e-34 | ORDERED | Relative age voice broke | *{younger than average … older than average}* |
| 35 | 6177 value 100 | Decrease | 4.37e-34 | BINARY | Medication for cholesterol, blood pressure or diabetes: “None of the above” | “None of the above” option not selected |
| 36 | 20151 | Decrease | 3.86e-33 | LINEAR | Forced vital capacity (FVC), best measure | - |
| 37 | 1468 (reference=3) | - | 4.96e-33 | MULTINOMIAL | Cereal type | “Oat cereal” |
| 38 | 137 | Increase | 3.99e-32 | ORDERED | Number of treatments/medications taken | *{quantity categories}* |
| 39 | 135 | Increase | 5.14e-32 | ORDERED | Number of self-reported non-cancer illnesses | *{3 quantity bands}* |
| 40 | 1428 (reference=3) | - | 6.15e-32 | MULTINOMIAL | Spread type | “Other type of spread/margarine” |
| 41 | 100010 | Increase | 3.64e-31 | ORDERED | Portion size | *{smaller … larger}* |
| 42 | 20511 | Increase | 6.96e-30 | ORDERED | Recent poor appetite or overeating | *{Not at all … nearly every day}* |
| 43 | 6144 value 5 | Decrease | 3.34e-29 | BINARY | I eat eggs, dairy, wheat, sugar | No |
| 44 | 1210 | Decrease | 4.03e-28 | BINARY | Snoring | Yes |
| 45 | 41204 value E668 | Increase | 1.39e-27 | BINARY | Diagnoses - secondary ICD10: other obesity | No |
| 46 | 3062 | Decrease | 2.13e-27 | LINEAR | Forced vital capacity (FVC) | - |
| 47 | 20111 value 100 | Decrease | 5.31e-27 | BINARY | Illnesses of siblings: “None of the above (group 1)” | “None of the above” option not selected for disease group 1 |
| 48 | 6153 value 2 | Increase | 2.97e-25 | BINARY | Medication for cholesterol, blood pressure, diabetes, or take exogenous hormones: blood pressure medication | No |
| 49 | 1448 (reference=3) | - | 8.04e-25 | MULTINOMIAL | Bread type | “Wholemeal or wholegrain” |
| 50 | 6159 value 7 | Increase | 1.76e-24 | BINARY | Pain type(s) experienced in last month: knee pain | No |
| 51 | 4079 | Increase | 2.00e-24 | LINEAR | Diastolic blood pressure, automated reading | *-* |
| 52 | 41200 value W401 | Increase | 4.59e-24 | BINARY | Operative procedures - main OPCS: primary total prosthetic replacement of knee joint using cement | No |
| 53 | 1970 | Decrease | 6.79e-24 | BINARY | Nervous feelings | No |
| 54 | 20086 value 10 | Increase | 1.12e-23 | BINARY | Type of special diet followed: low calorie | No |
| 55 | 1548 | Increase | 1.65e-23 | ORDERED | Variation in diet | *{Never/rarely … often}* |
| 56 | 30000 |  |  |  |  |  |
| 57 | 3144 | Increase | 2.40e-22 | LINEAR | Heel Broadband ultrasound attenuation, direct entry | - |
| 58 | 20111 value 8 | Increase | 6.34e-22 | BINARY | Illnesses of siblings: high blood pressure | No |
| 59 | 20111 value 9 | Increase | 2.81e-21 | BINARY | Illnesses of siblings: diabetes | No |
| 60 | 20003 value 1141194794 | Increase | 7.41e-21 | BINARY | Treatment/medication code: bendroflumethiazide | No |
| 61 | 30530 | Increase | 1.16e-20 | LINEAR | Sodium in urine | - |
| 62 | 20002 value 1465 | Increase | 1.42e-20 | BINARY | on-cancer illness code, self-reported: osteoarthritis | No |
| 63 | 41202 value E668 | Increase | 2.18e-20 | BINARY | Diagnoses - main ICD10: other obesity | No |
| 64 | 4101 | Increase | 2.79e-20 | LINEAR | Heel broadband ultrasound attenuation (left) | *-* |
| 65 | 1289 | Increase | 4.46e-20 | ORDERED | Cooked vegetable intake | *{3 quantity bands}* |
| 66 | 6159 value 6 | Increase | 5.33e-20 | BINARY | Pain type(s) experienced in last month: hip pain | No |
| 67 | 2492 | Increase | 7.50e-20 | BINARY | Taking other prescription medications | No |
| 68 | 6146 value 100 | Decrease | 1.46e-19 | BINARY | Attendance/disability/mobility allowance: “None of the above” | “None of the above” option not selected |
| 69 | 4120 | Increase | 1.81e-19 | LINEAR | Heel broadband ultrasound attenuation (right) | - |
| 70 | 20116 (reference=0) | - | 4.94e-19 | MULTINOMIAL | Smoking status | Never |
| 71 | 20004 value 1455 | Increase | 1.16e-18 | BINARY | Operation code: cholecystectomy/gall bladder removal | No |
| 72 | 1309 | Increase | 1.94e-18 | ORDERED | Fresh fruit intake | *{3 quantity bands}* |
| 73 | 41202 value M179 | Increase | 2.18e-18 | BINARY | Diagnoses - main ICD10: gonarthrosis, unspecified | No |
| 74 | 100490 | Decrease | 3.64e-18 | ORDERED | Intake of sugar added to tea | *{half … 3+}* |
| 75 | 6177 value 1 | Increase | 9.31e-18 | BINARY | Medication for cholesterol, blood pressure or diabetes: cholesterol lowering medication | No |
| 76 | 2654 (reference=7) | - | 1.01e-17 | MULTINOMIAL | Non-butter spread type details | “Polyunsaturated/sunflower oil based spread” |
| 77 | 6146 value 3 | Increase | 1.22e-17 | BINARY | Attendance/disability/mobility allowance: blue badge | No |
| 78 | 30140 | Increase | 2.50e-17 | LINEAR | Neutrophill count | - |
| 79 | 4728 | Increase | 7.53e-17 | BINARY | Leg pain on walking | No |
| 80 | 4548 | Increase | 9.04e-17 | ORDERED | Health satisfaction | *{Extremely happy … extremely unhappy}* |
| 81 | 20150 | Decrease | 1.21e-16 | LINEAR | Forced expiratory volume in 1-second (FEV1), best measure | - |
| 82 | 30500 | Increase | 1.52e-16 | LINEAR | Microalbumin in urine | *-* |
| 83 | 1757 | Increase | 1.85e-16 | ORDERED | Facial ageing | *{younger than you are … older than you are}* |
| 84 | 1249 | Decrease | 2.38e-16 | ORDERED | Past tobacco smoking | *{Smoked on most or all days … never smoked}* |
| 85 | 1070 | Increase | 3.52e-16 | ORDERED | Time spent watching television (TV) | *{3 time bands}* |
| 86 | 41244 value 100 | Increase | 1.23e-15 | BINARY | Intended management of patient (recoded): one or more nights hospital stay | No |
| 87 | 738 | Decrease | 1.25e-15 | ORDERED | Average total household income before tax | *{Less than 18,000 … greater than 100,000}* |
| 88 | 41247 value 1000 | Increase | 1.52e-15 | BINARY | Patient classification on admission (recoded): Inpatient | No |
| 89 | 20003 value 1140860806 | Increase | 2.00e-15 | BINARY | Treatment/medication code: ramipril | No |
| 90 | 20003 value 1140874744 | Increase | 2.48e-15 | BINARY | Treatment/medication code: gliclazide | No |
| 91 | 136 | Increase | 3.71e-15 | ORDERED | Number of operations, self-reported | *{3 quantity bands}* |
| 92 | 943 | Decrease | 3.74e-15 | ORDERED | Frequency of stair climbing in last 4 weeks | *{none … more than 20 times a day}* |
| 93 | 41202 value G560 | Increase | 9.53e-15 | BINARY | Diagnoses - main ICD10: carpal tunnel syndrome | *No* |
| 94 | 20003 value 1140909708 | Increase | 1.00e-14 | BINARY | Treatment/medication code: furosemide | No |
| 95 | 30510 | Increase | 1.00e-14 | LINEAR | Creatinine (enzymatic) in urine | *-* |
| 96 | 20154 | Decrease | 1.25e-14 | LINEAR | Forced expiratory volume in 1-second (FEV1), predicted percentage | *-* |
| 97 | 30150 | Increase | 1.69e-14 | ORDERED | Eosinophill count | *{3 count bands}* |
| 98 | 4717 | Increase | 1.89e-14 | BINARY | Shortness of breath walking on level ground | No |
| 99 | 1369 | Decrease | 2.79e-14 | ORDERED | Beef intake | *{Never … once or more daily}* |
| 100 | 6146 value 2 | Increase | 2.86e-14 | BINARY | Attendance/disability/mobility allowance: disability living allowance | No |
| 101 | 30120 | Increase | 2.93e-14 | LINEAR | Lymphocyte count | *-* |
| 102 | 1408 | Decrease | 3.15e-14 | ORDERED | Cheese intake | *{never … once or more daily}* |
| 103 | 41204 value Z921 | Increase | 3.32e-14 | BINARY | Diagnoses - secondary ICD10: personal history of long-term (current) use of anticoagulants | No |
| 104 | 41200 value X998 | Increase | 3.95e-14 | BINARY | Operative procedures - main OPCS: no procedure performed | No |
| 105 | 41200 value A651 | Increase | 6.78e-14 | BINARY | Operative procedures - main OPCS: carpal tunnel release | No |
| 106 | 20003 value 1140879802 | Increase | 1.67e-13 | BINARY | Treatment/medication code: amlodipine | No |
| 107 | 30100 | Increase | 2.00e-13 | LINEAR | Mean platelet (thrombocyte) volume | *-* |
| 108 | 6138 value 1 | Decrease | 2.08e-13 | BINARY | Qualifications: college or university degree | No |
| 109 | 20003 value 1141171646 | Increase | 2.37e-13 | BINARY | Treatment/medication code: pioglitazone | No |
| 110 | 41202 value L031 | Increase | 3.85e-13 | BINARY | Diagnoses - main ICD10: cellulitis of other parts of limb | No |
| 111 | 41249 value 1001 | Increase | 4.96e-13 | BINARY | Methods of admission to hospital (recoded): elective admission: Waiting List | No |
| 112 | 41231 value 1 | Increase | 7.89e-13 | BINARY | Hospital episode type: General episode | No |
| 113 | 20004 value 1319 | Increase | 9.27e-13 | BINARY | Operation code: knee replacement/revision | No |
| 114 | 20003 value 1140883066 | Increase | 1.21e-12 | BINARY | Treatment/medication code: insulin product | No |
| 115 | 1628 | Increase | 1.70e-12 | ORDERED | Alcohol intake versus 10 years previously | *{More nowadays … less nowadays}* |
| 116 | 20414 | Decrease | 1.96e-12 | ORDERED | Frequency of drinking alcohol | *{Never … 4 or more times a week}* |
| 117 | 41204 value Z966 | Increase | 2.11e-12 | BINARY | Diagnoses - secondary ICD10: presence of orthopaedic joint implants | No |
| 118 | 22408 | Increase | 2.17e-12 | LINEAR | Abdominal subcutaneous adipose tissue volume (ASAT) | - |
| 119 | 41204 value I209 | Increase | 2.45e-12 | BINARY | Diagnoses - secondary ICD10: angina pectoris, unspecified | No |
| 120 | 100920 (reference=2102) | - | 2.56e-12 | MULTINOMIAL | Type milk consumed | Semi-skimmed |
| 121 | 20459 | Increase | 2.88e-12 | ORDERED | General happiness with own health | *{Extremely happy … extremely unhappy}* |
| 122 | 2139 | Decrease | 3.67e-12 | LINEAR | Age first had sexual intercourse | *-* |
| 123 | 2804 | Decrease | 4.23e-12 | LINEAR | Age when last used oral contraceptive pill | - |
| 124 | 100370 | Decrease | 5.13e-12 | ORDERED | Intake of sugar added to coffee | *{half … 3+}* |
| 125 | 20003 value 1140868226 | Increase | 5.81e-12 | BINARY | Treatment/medication code: aspirin | No |
| 126 | 41253 value 6 | Increase | 7.71e-12 | BINARY | Source of inpatient record: English HES Data | No |
| 127 | 20110 value 9 | Increase | 8.27e-12 | BINARY | Illnesses of mother: diabetes | No |
| 128 | 1807 | Decrease | 9.15e-12 | LINEAR | Father's age at death | - |
| 129 | 1990 | Decrease | 1.02e-11 | BINARY | Tense / 'highly strung' | No |
| 130 | 6138 value 2 | Decrease | 1.27e-11 | BINARY | Qualifications: A levels/AS levels or equivalent | No |
| 131 | 41204 value G473 | Increase | 1.32e-11 | BINARY | Diagnoses - secondary ICD10: Sleep apnoea | No |
| 132 | 20003 value 1140861958 | Increase | 1.43e-11 | BINARY | Treatment/medication code: simvastatin | No |
| 133 | 4080 | Increase | 1.51e-11 | LINEAR | Systolic blood pressure, automated reading | - |
| 134 | 20107 value 9 | Increase | 1.68e-11 | BINARY | Illnesses of father: diabetes | No |
| 135 | 6154 value 1 | Increase | 1.95e-11 | BINARY | Medication for pain relief, constipation, heartburn: aspirin | No |
| 136 | 3063 | Decrease | 2.06e-11 | LINEAR | Forced expiratory volume in 1-second (FEV1) | - |
| 137 | 3148 | Increase | 3.08e-11 | LINEAR | Heel bone mineral density (BMD) | - |
| 138 | 41248 value 1000 | Increase | 3.27e-11 | BINARY | Destinations on discharge from hospital (recoded): usual place of residence | No |
| 139 | 41202 value M171 | Increase | 3.33e-11 | BINARY | Diagnoses - main ICD10: other primary gonarthrosis | No |
| 140 | 41245 value 1840 | Increase | 3.38e-11 | BINARY | Main speciality of consultant (recoded): trauma and orthopaedics | No |
| 141 | 4105 | Increase | 3.55e-11 | LINEAR | Heel bone mineral density (BMD) (left) | - |
| 142 | 41204 value E149 | Increase | 3.57e-11 | BINARY | Diagnoses - secondary ICD10: unspecified diabetes mellitus without complications | No |
| 143 | 4106 | Increase | 4.91e-11 | LINEAR | Heel bone mineral density (BMD) T-score, automated (left) | - |
| 144 | 4104 | Increase | 4.91e-11 | LINEAR | Heel quantitative ultrasound index (QUI), direct entry (left) | - |
| 145 | 41204 value L031 | Increase | 5.02e-11 | BINARY | Diagnoses - secondary ICD10: cellulitis of other parts of limb | No |
| 146 | 20002 value 1223 | Increase | 6.65e-11 | BINARY | Non-cancer illness code, self-reported: type 2 diabetes | No |
| 147 | 20004 value 1318 | Increase | 7.00e-11 | BINARY | Operation code: hip replacement/revision | No |
| 148 | 78 | Increase | 7.16e-11 | LINEAR | Heel bone mineral density (BMD) T-score, automated | - |
| 149 | 3147 | Increase | 7.17e-11 | LINEAR | Heel quantitative ultrasound index (QUI), direct entry | - |
| 150 | 6164 value 4 | Decrease | 8.20e-11 | BINARY | Types of physical activity in last 4 weeks: light DIY | No |
| 151 | 1299 | Increase | 1.03e-10 | ORDERED | Salad / raw vegetable intake | *{3 quantity bands}* |
| 152 | 20003 value 1140879778 | Increase | 1.14e-10 | BINARY | Treatment/medication code: doxazosin | No |
| 153 | 41250 value 1000 | Increase | 1.22e-10 | BINARY | Methods of discharge from hospital (recoded): discharged on clinical advice/consent | No |
| 154 | 845 | Decrease | 1.36e-10 | ORDERED | Age completed full time education | *{3 age bands}* |
| 155 | 20161 | Increase | 1.61e-10 | LINEAR | Pack years of smoking | - |
| 156 | 20022 | Increase | 1.66e-10 | LINEAR | Birth weight | - |
| 157 | 41210 value Z942 | Increase | 1.85e-10 | BINARY | Operative procedures - secondary OPCS: right sided operation | No |
| 158 | 6138 value 100 | Increase | 2.02e-10 | BINARY | Qualifications: “None of the above“ | “None of the above” option not selected |
| 159 | 20162 | Increase | 2.04e-10 | LINEAR | Pack years adult smoking as proportion of life span exposed to smoking | - |
| 160 | 100160 | Increase | 2.37e-10 | ORDERED | Low calorie drink intake | *{half … 6+}* |
| 161 | 6152 value 5 | Increase | 2.43e-10 | BINARY | Blood clot, DVT, bronchitis, emphysema, asthma, rhinitis, eczema, allergy diagnosed by doctor: Blood clot in the leg (DVT) | No |
| 162 | 4123 | Increase | 2.48e-10 | LINEAR | Heel quantitative ultrasound index (QUI), direct entry (right) | - |
| 163 | 4125 | Increase | 2.49e-10 | LINEAR | Heel bone mineral density (BMD) T-score, automated (right) | - |
| 164 | 4124 | Increase | 2.58e-10 | LINEAR | Heel bone mineral density (BMD) (right) | - |
| 165 | 20002 value 1162 | Increase | 2.69e-10 | BINARY | Non-cancer illness code, self-reported: cholelithiasis/gall stones | No |
| 166 | 41246 value 2580 | Increase | 2.69e-10 | BINARY | Treatment speciality of consultant (recoded): trauma & orthopaedics | *No* |
| 167 | 20160 | Increase | 3.32e-10 | BINARY | Ever smoked | No |
| 168 | 41210 value Z943 | Increase | 4.14e-10 | BINARY | Operative procedures - secondary OPCS: laterality of operation - Left sided operation | No |
| 169 | 20110 value 8 | Increase | 4.62e-10 | BINARY | Illnesses of mother: high blood pressure | No |
| 170 | 6148 value 1 | Increase | 5.23e-10 | BINARY | Eye problems/disorders: diabetes related eye disease | No |
| 171 | 6142 value 4 | Increase | 6.00e-10 | BINARY | Current employment status: unable to work because of sickness or disability | No |
| 172 | 22410 | Increase | 6.42e-10 | LINEAR | Total trunk fat | - |
| 173 | 41204 value E039 | Increase | 6.46e-10 | BINARY | Diagnoses - secondary ICD10: hypothyroidism, unspecified | No |
| 174 | 20544 value 13 | Increase | 7.39e-10 | BINARY | Mental health problems ever diagnosed by a professional: Psychological over-eating or binge-eating | No |
| 175 | 22409 | Increase | 7.79e-10 | LINEAR | Total thigh muscle volume | - |
| 176 | 2296 | Increase | 8.16e-10 | ORDERED | Falls in the last year | *{no falls … more than one fall}* |
| 177 | 22405 | Increase | 8.22e-10 | LINEAR | Anterior thigh lean muscle volume (left) | - |
| 178 | 25741 | Increase | 8.92e-10 | LINEAR | Mean rfMRI head motion averaged across space and time points | - |
| 179 | 20003 value 1141152998 | Increase | 9.65e-10 | BINARY | Treatment/medication code: irbesartan | No |
| 180 | 2010 | Decrease | 9.73e-10 | BINARY | Suffer from 'nerves' | No |
| 181 | 41251 value 1000 | Increase | 1.25e-09 | BINARY | Sources of admission to hospital (recoded): ): usual place of residence | No |
| 182 | 22406 | Increase | 1.27e-09 | LINEAR | Posterior thigh lean muscle volume (left) | - |
| 183 | 20003 value 1140860696 | Increase | 1.40e-09 | BINARY | Treatment/medication code: lisinopril | No |
| 184 | 6177 value 3 | Increase | 1.60e-09 | BINARY | Medication for cholesterol, blood pressure or diabetes: insulin | No |
| 185 | 20002 value 1094 | Increase | 1.91e-09 | BINARY | Non-cancer illness code, self-reported: deep venous thrombosis | No |
| 186 | 22427 | Increase | 2.18e-09 | LINEAR | Body surface area | - |
| 187 | 41204 value Z864 | Increase | 2.71e-09 | BINARY | Diagnoses - secondary ICD10: Personal history of psychoactive substance abuse | No |
| 188 | 41202 value G473 | Increase | 3.37e-09 | BINARY | Diagnoses - main ICD10: sleep apnoea | No |
| 189 | 41245 value 1310 | Increase | 3.85e-09 | BINARY | Main speciality of consultant (recoded): general medicine | No |
| 190 | 6149 value 6 | Increase | 4.01e-09 | BINARY | Mouth/teeth dental problems: dentures | No |
| 191 | 1359 | Increase | 5.90e-09 | ORDERED | Poultry intake | *{never … 5-6 times a week}* |
| 192 | 6164 value 5 | Decrease | 6.15e-09 | BINARY | Types of physical activity in last 4 weeks: heavy DIY | No |
| 193 | 1598 | Increase | 6.45e-09 | ORDERED | Average weekly spirits intake | *{3 intake bands}* |
| 194 | 41204 value J459 | Increase | 6.73e-09 | BINARY | Diagnoses - secondary ICD10: asthma, unspecified | No |
| 195 | 41204 value I48 | Increase | 8.34e-09 | BINARY | Diagnoses - secondary ICD10: atrial fibrillation and flutter | No |
| 196 | 41253 value 18 | Increase | 8.61e-09 | BINARY | Source of inpatient record: UK HES In Patient (2015 format) | No |
| 197 | 20004 value 1501 | Increase | 8.84e-09 | BINARY | Operation code: carpal tunnel surgery | No |
| 198 | 20003 value 1140888560 | Increase | 1.02e-08 | BINARY | Treatment/medication code: perindopril | No |
| 199 | 189 | Increase | 1.05e-08 | LINEAR | Townsend deprivation index at recruitment | - |
| 200 | 22404 | Increase | 1.27e-08 | LINEAR | Posterior thigh lean muscle volume (right) | - |
| 201 | 20003 value 1140875408 | Increase | 1.28e-08 | BINARY | Treatment/medication code: allopurinol | No |
| 202 | 41204 value I259 | Increase | 1.39e-08 | BINARY | Diagnoses - secondary ICD10: chronic ischaemic heart disease, unspecified | No |
| 203 | 41204 value H360 | Increase | 1.43e-08 | BINARY | Diagnoses - secondary ICD10: diabetic retinopathy | No |
| 204 | 6162 value 2 | Decrease | 1.47e-08 | BINARY | Types of transport used (excluding work): walk | No |
| 205 | 20003 value 1141168684 | Increase | 1.53e-08 | BINARY | Treatment/medication code: xenical 120mg capsule | No |
| 206 | 41200 value W381 | Increase | 1.60e-08 | BINARY | Operative procedures - main OPCS: primary total prosthetic replacement of hip joint not using cement | No |
| 207 | 41246 value 1490 | Increase | 2.16e-08 | BINARY | Treatment speciality of consultant (recoded): general medicine | No |
| 208 | 2764 | Decrease | 2.33e-08 | LINEAR | Age at last live birth | - |
| 209 | 6033 | Decrease | 2.37e-08 | LINEAR | Maximum heart rate during fitness test | - |
| 210 | 41204 value Z922 | Increase | 2.54e-08 | BINARY | Diagnoses - secondary ICD10: Personal history of long-term (current) use of other medicaments | No |
| 211 | 41200 value J183 | Increase | 2.91e-08 | BINARY | Operative procedures - main OPCS: Total cholecystectomy NEC | No |
| 212 | 30260 | Increase | 2.96e-08 | LINEAR | Mean reticulocyte volume | - |
| 213 | 1329 | Increase | 3.30e-08 | ORDERED | Oily fish intake | *{never … once or more daily}* |
| 214 | 20002 value 1226 | Increase | 3.55e-08 | BINARY | "Non-cancer illness code, self-reported: hypothyroidism/myxedema | No |
| 215 | 2754 | Decrease | 3.81e-08 | LINEAR | Age at first live birth | - |
| 216 | 1518 | Decrease | 3.96e-08 | ORDERED | Hot drink temperature | *{Very hot ... warm}* |
| 217 | 1797 | Decrease | 4.01e-08 | BINARY | Father still alive | No |
| 218 | 30520 | Increase | 4.88e-08 | LINEAR | Potassium in urine | - |
| 219 | 41200 value G331 | Increase | 5.02e-08 | BINARY | Operative procedures - main OPCS: bypass of stomach by anastomosis of stomach to jejunum NEC | No |
| 220 | 20003 value 1140866738 | Increase | 5.60e-08 | BINARY | Treatment/medication code: atenolol | No |
| 221 | 41204 value E114 | Increase | 6.67e-08 | BINARY | Diagnoses - secondary ICD10: insulin-dependent diabetes mellitus, with neurological complications | No |
| 222 | 41246 value 1530 | Increase | 6.72e-08 | BINARY | Treatment speciality of consultant (recoded): general surgery | No |
| 223 | 6160 value 2 | Decrease | 7.84e-08 | BINARY | Leisure/social activities: pub or social club | No |
| 224 | 2080 | Increase | 8.10e-08 | ORDERED | Frequency of tiredness / lethargy in last 2 weeks | *{Not at all ... nearly every day}* |
| 225 | 20004 value 1479 | Increase | 9.62e-08 | BINARY | Operation code: varicose vein surgery | No |
| 226 | 6149 value 1 | Decrease | 9.88e-08 | BINARY | Mouth/teeth dental problems: mouth ulcers | No |
| 227 | 1220 | Increase | 1.07e-07 | ORDERED | Daytime dozing / sleeping (narcolepsy) | *{Never/rarely ... often}* |
| 228 | 20111 value 1 | Increase | 1.30e-07 | BINARY | Illnesses of siblings: heart disease | No |
| 229 | 41249 value 2001 | Increase | 1.41e-07 | BINARY | Methods of admission to hospital (recoded): emergency admission: A&E | No |
| 230 | 6164 value 100 | Increase | 1.62e-07 | BINARY | Types of physical activity in last 4 weeks: “None of the above” | “None of the above” option not selected |
| 231 | 20003 value 1140888266 | Increase | 1.66e-07 | BINARY | Treatment/medication code: warfarin | No |
| 232 | 20004 value 1496 | Increase | 1.79e-07 | BINARY | Operation code: knee surgery (not replacement) | No |
| 233 | 41202 value M7986 | Increase | 1.81e-07 | BINARY | Diagnoses - main ICD10: other specified soft tissue disorders (Lower leg) | No |
| 234 | 41246 value 1160 | Increase | 2.20e-07 | BINARY | Treatment speciality of consultant (recoded): cardiology | No |
| 235 | 41210 value Z846 | Increase | 2.23e-07 | BINARY | Operative procedures - secondary OPCS: Knee joint | No |
| 236 | 41204 value M179 | Increase | 2.25e-07 | BINARY | Diagnoses - secondary ICD10: gonarthrosis, unspecified | No |
| 237 | 41249 value 2002 | Increase | 2.43e-07 | BINARY | Methods of admission to hospital (recoded): emergency admission: GP | No |
| 238 | 826 | Increase | 2.52e-07 | ORDERED | Job involves shift work | *{never/rarely … always}* |
| 239 | 3546 | Decrease | 2.63e-07 | LINEAR | Age last used hormone-replacement therapy (HRT) | - |
| 240 | 100380 | Increase | 2.71e-07 | ORDERED | Intake of artificial sweetener added to coffee | *{half … 3+}* |
| 241 | 41204 value E780 | Increase | 2.88e-07 | BINARY | Diagnoses - secondary ICD10: pure hypercholesterolaemia | No |
| 242 | 20003 value 1140923346 | Increase | 3.02e-07 | BINARY | Treatment/medication code: co-codamol | No |
| 243 | 41204 value Z867 | Increase | 3.04e-07 | BINARY | Diagnoses - secondary ICD10: personal history of diseases of the circulatory system | No |
| 244 | 1239 | Increase | 3.13e-07 | ORDERED | Current tobacco smoking | *{no … yes, on most or all days}* |
| 245 | 20004 value 1369 | Decrease | 3.31e-07 | BINARY | Operation code: lumpectomy | No |
| 246 | 41235 | Increase | 3.96e-07 | ORDERED | Spells in hospital | *{3 frequency bands}* |
| 247 | 100250 | Increase | 4.28e-07 | ORDERED | Instant coffee intake | *{half … 6+}* |
| 248 | 6150 value 2 | Increase | 5.34e-07 | BINARY | Vascular/heart problems diagnosed by doctor: angina | No |
| 249 | 41204 value J449 | Increase | 5.39e-07 | BINARY | Diagnoses - secondary ICD10: Chronic obstructive pulmonary disease, unspecified | No |
| 250 | 20002 value 1074 | Increase | 5.67e-07 | BINARY | Non-cancer illness code, self-reported: angina | No |
| 251 | 20001 value 1002 | Decrease | 5.83e-07 | BINARY | Cancer code, self-reported: breast cancer | No |
| 252 | 41204 value I258 | Increase | 6.22e-07 | BINARY | Diagnoses - secondary ICD10: Other forms of chronic ischaemic heart disease | No |
| 253 | 23307 | Increase | 6.23e-07 | LINEAR | Pelvis bone area | - |
| 254 | 20003 value 1140864992 | Increase | 6.71e-07 | BINARY | Treatment/medication code: tramadol | No |
| 255 | 2473 | Increase | 7.06e-07 | BINARY | Other serious medical condition/disability diagnosed by doctor | No |
| 256 | 2020 | Increase | 7.24e-07 | BINARY | Loneliness, isolation | No |
| 257 | 41232 value 10 | Increase | 7.76e-07 | BINARY | Administrative and legal statuses: NHS patient: not formally detained |  |
| 258 | 41204 value E113 | Increase | 8.01e-07 | BINARY | Diagnoses - secondary ICD10: With ophthalmic complications | No |
| 259 | 1349 | Decrease | 8.50e-07 | ORDERED | Processed meat intake | *{never … once or more daily}* |
| 260 | 6146 value 1 | Increase | 1.00e-06 | BINARY | Attendance/disability/mobility allowance: attendance allowance | No |
| 261 | 41210 value W581 | Increase | 1.03e-06 | BINARY | Operative procedures - secondary OPCS: primary resurfacing arthroplasty of joint | No |
| 262 | 41245 value 1350 | Increase | 1.03e-06 | BINARY | Main speciality of consultant (recoded): general surgery | No |
| 263 | 41202 value I802 | Increase | 1.03e-06 | BINARY | Diagnoses - main ICD10: phlebitis and thrombophlebitis of other deep vessels of lower extremities | No |
| 265 | 20107 value 1 | Increase | 1.07e-06 | BINARY | Illnesses of father: heart disease | No |
| 264 | 41245 value 1070 | Increase | 1.18e-06 | BINARY | Main speciality of consultant (recoded): cardiology | No |
| 266 | 20003 value 1141168680 | Increase | 1.26e-06 | BINARY | Treatment/medication code: orlistat | No |
| 267 | 2395 (reference=1) | - | 1.33e-06 | MULTINOMIAL | Hair/balding pattern | Full head of hair |
| 268 | 1980 | Decrease | 1.34e-06 | BINARY | Worrier / anxious feelings | No |
| 269 | 20003 value 1140860840 | Increase | 1.37e-06 | BINARY | Treatment/medication code: nitrolingual 400micrograms spray | No |
| 270 | 20003 value 1141156836 | Increase | 1.39e-06 | BINARY | Treatment/medication code: candesartan cilexetil | No |
| 271 | 41204 value M5499 | Increase | 1.40e-06 | BINARY | Diagnoses - secondary ICD10: Dorsalgia, unspecified (Site unspecified) | No |
| 272 | 6154 value 6 | Increase | 1.47e-06 | BINARY | Medication for pain relief, constipation, heartburn: Laxatives | No |
| 273 | 20003 value 1140888552 | Increase | 1.57e-06 | BINARY | Treatment/medication code: enalapril | No |
| 274 | 20002 value 1466 | Increase | 1.66e-06 | BINARY | Non-cancer illness code, self-reported: gout | No |
| 275 | 22403 | Increase | 1.67e-06 | LINEAR | Anterior thigh lean muscle volume (right) | - |
| 276 | 41204 value L309 | Increase | 1.83e-06 | BINARY | Diagnoses - secondary ICD10: Dermatitis, unspecified | No |
| 277 | 6152 value 8 | Increase | 1.97e-06 | BINARY | Blood clot, DVT, bronchitis, emphysema, asthma, rhinitis, eczema, allergy diagnosed by doctor: Asthma | No |
| 278 | 41204 value I839 | Increase | 1.97e-06 | BINARY | Diagnoses - secondary ICD10: Varicose veins of lower extremities without ulcer or inflammation | No |
| Bonferroni P value threshold: 2.18x10^-6^ | | | | | | |
| 279 | 20003 value 1141177600 | Increase | 2.21e-06 | BINARY | Treatment/medication code: rosiglitazone | No |
| 280 | 41202 value K802 | Increase | 2.22e-06 | BINARY | Diagnoses - main ICD10: calculus of gallbladder without cholecystitis | No |
| 281 | 20003 value 1141191044 | Increase | 2.32e-06 | BINARY | Treatment/medication code: levothyroxine sodium | No |
| Correlation-based FWER P value threshold: 2.55x10^-6^ | | | | | | |
| 282 | 20002 value 1111 | Increase | 2.66e-06 | BINARY | Non-cancer illness code, self-reported: asthma | No |
| 283 | 41204 value I252 | Increase | 2.79e-06 | BINARY | Diagnoses - secondary ICD10: Old myocardial infarction | No |
| 284 | 41204 value Z538 | Increase | 2.82e-06 | BINARY | Diagnoses - secondary ICD10: Procedure not carried out for other reasons | No |
| 285 | 41200 value G303 | Increase | 3.05e-06 | BINARY | Operative procedures - main OPCS: partitioning of stomach using band | No |
| 286 | 1787 | Increase | 3.15e-06 | BINARY | Maternal smoking around birth | No |
| 287 | 25742 | Increase | 3.20e-06 | LINEAR | Mean tfMRI head motion averaged across space and time points | - |
| 288 | 25743 | Increase | 3.47e-06 | LINEAR | Inverted temporal signal-to-noise ratio in pre-processed rfMRI | - |
| 289 | 22608 value 1 | Increase | 3.59e-06 | BINARY | Workplace very hot | No |
| 290 | 41200 value T242 | Increase | 3.66e-06 | BINARY | Operative procedures - main OPCS: Repair of umbilical hernia using insert of prosthetic material | No |
| 291 | 41204 value M1399 | Increase | 3.70e-06 | BINARY | Diagnoses - secondary ICD10: Arthritis, unspecified (Site unspecified) | No |
| 292 | 41204 value Z951 | Increase | 3.97e-06 | BINARY | Diagnoses - secondary ICD10: presence of aortocoronary bypass graft | No |
| 293 | 41202 value M169 | Increase | 4.18e-06 | BINARY | Diagnoses - main ICD10: Coxarthrosis, unspecified | No |
| 294 | 2784 | Decrease | 4.48e-06 | BINARY | Ever taken oral contraceptive pill | No |
| 295 | 6150 value 1 | Increase | 4.90e-06 | BINARY | Vascular/heart problems diagnosed by doctor: heart attack | No |
| 296 | 20117 (reference=2) | - | 5.62e-06 | MULTINOMIAL | Alcohol drinker status | Current |
| 297 | 41200 value W901 | Increase | 5.78e-06 | BINARY | Operative procedures - main OPCS: Aspiration of joint | No |
| 298 | 22607 value 1 | Increase | 5.81e-06 | BINARY | Workplace very cold | No |
| 299 | 20004 value 1095 | Increase | 5.90e-06 | BINARY | Operation code: coronary artery bypass grafts (cabg) | No |
| 300 | 20110 value 100 | Decrease | 6.47e-06 | BINARY | Illnesses of mother: “None of the above (group 1)’ | “None of the above (group 1)” option not selected |
| 301 | 20002 value 1075 | Increase | 6.71e-06 | BINARY | Non-cancer illness code, self-reported: heart attack/myocardial infarction | No |
| 302 | 1588 | Decrease | 6.78e-06 | ORDERED | Average weekly beer plus cider intake | *{3 quantity bands}* |
| 303 | 30080 | Decrease | 6.90e-06 | LINEAR | Platelet count | - |
| 304 | 100390 | Decrease | 7.06e-06 | BINARY | Tea consumed | No |
| 305 | 30190 | Decrease | 7.56e-06 | LINEAR | Monocyte percentage | - |
| 306 | 41202 value E669 | Increase | 7.74e-06 | BINARY | Diagnoses - main ICD10: Obesity, unspecified | No |
| 307 | 6149 value 4 | Increase | 8.29e-06 | BINARY | Mouth/teeth dental problems: loose teeth | No |
| 308 | 20002 value 1309 | Decrease | 8.32e-06 | BINARY | Non-cancer illness code, self-reported: osteoporosis | No |
| 309 | 41204 value I517 | Increase | 8.34e-06 | BINARY | Diagnoses - secondary ICD10: cardiomegaly | No |
| 310 | 41200 value W371 | Increase | 8.38e-06 | BINARY | Operative procedures - main OPCS: primary total prosthetic replacement of hip joint using cement | No |
| 311 | 41244 value 200 | Increase | 9.34e-06 | BINARY | Intended management of patient (recoded): no overnight hospital stay | No |
| 312 | 6159 value 4 | Increase | 9.88e-06 | BINARY | Pain type(s) experienced in last month: Back pain | No |
| 313 | 20003 value 1140866116 | Increase | 1.03e-05 | BINARY | Treatment/medication code: frusemide | No |
| 314 | 1180 | Decrease | 1.04e-05 | ORDERED | Morning/evening person (chronotype) | *{Definitely a 'morning' person … Definitely an 'evening' person}* |
| 315 | 41202 value K805 | Increase | 1.13e-05 | BINARY | Diagnoses - main ICD10: Calculus of bile duct without cholangitis or cholecystitis | No |
| 316 | 20002 value 1123 | Increase | 1.16e-05 | BINARY | Non-cancer illness code, self-reported: sleep apnoea | No |
| 317 | 2744 | Increase | 1.16e-05 | ORDERED | Birth weight of first child | *{3 weight bands}* |
| 318 | 23318 | Increase | 1.19e-05 | LINEAR | Legs combined bone area | - |
| 319 | 3526 | Decrease | 1.23e-05 | LINEAR | Mother's age at death | - |
| 320 | 6159 value 1 | Decrease | 1.24e-05 | BINARY | Pain type(s) experienced in last month: headache | No |
| 321 | 6164 value 1 | Decrease | 1.26e-05 | BINARY | Types of physical activity in last 4 weeks: walking for pleasure (not as a means of transport) | No |
| 322 | 20003 value 1141146234 | Increase | 1.27e-05 | BINARY | Treatment/medication code: atorvastatin | No |
| 323 | 20107 value 100 | Decrease | 1.30e-05 | BINARY | Illnesses of father: “None of the above (group 1)” | “None of the above (group 1)” option not selected |
| 324 | 100180 | Decrease | 1.49e-05 | ORDERED | Squash intake | *{half … 6+}* |
| 325 | 1528 | Increase | 1.65e-05 | ORDERED | Water intake | *{3 quantity bands}* |
| 326 | 90144 | Increase | 1.65e-05 | ORDERED | Fraction acceleration <= 600 milli-gravities | *{3 fraction bands}* |
| 327 | 6138 value 3 | Decrease | 1.68e-05 | BINARY | Qualifications: O levels/GCSEs or equivalent | No |
| 328 | 1379 | Decrease | 1.70e-05 | ORDERED | Lamb/mutton intake | *{never … once or more daily}* |
| 329 | 6153 value 3 | Increase | 1.76e-05 | BINARY | Medication for cholesterol, blood pressure, diabetes, or take exogenous hormones: insulin | No |
| 330 | 6139 value 3 | Decrease | 1.80e-05 | BINARY | Gas or solid-fuel cooking/heating | “An open solid fuel fire that you use regularly in winter time” |
| 331 | 6159 value 100 | Decrease | 1.84e-05 | BINARY | Pain type(s) experienced in last month: “None of the above“ | “None of the above” option not selected |
| 332 | 41204 value Z880 | Increase | 1.91e-05 | BINARY | Diagnoses - secondary ICD10: Personal history of allergy to penicillin | No |
| 333 | 30210 | Increase | 1.92e-05 | LINEAR | Eosinophill percentage | - |
| 334 | 41204 value I500 | Increase | 1.92e-05 | BINARY | Diagnoses - secondary ICD10: Congestive heart failure | No |
| 335 | 41204 value M751 | Increase | 2.08e-05 | BINARY | Diagnoses - secondary ICD10: Rotator cuff syndrome | No |
| 336 | 1737 | Increase | 2.10e-05 | ORDERED | Childhood sunburn occasions | *{3 quantity bands}* |
| 337 | 6153 value 100 | Decrease | 2.10e-05 | BINARY | Medication for cholesterol, blood pressure, diabetes, or take exogenous hormones: None of the above | “None of the above” option not selected |
| 338 | 20003 value 1140874420 | Increase | 2.22e-05 | BINARY | Treatment/medication code: quinine | No |
| 339 | 23230 | Increase | 2.25e-05 | LINEAR | Legs BMC (bone mineral content) | - |
| 340 | 41204 value L97 | Increase | 2.26e-05 | BINARY | Diagnoses - secondary ICD10: Ulcer of lower limb, not elsewhere classified | No |
| 341 | 41210 value Y767 | Increase | 2.26e-05 | BINARY | Operative procedures - secondary OPCS: Arthroscopic approach to joint | No |
| 342 | 41204 value Z871 | Increase | 2.29e-05 | BINARY | Diagnoses - secondary ICD10: Personal history of diseases of the digestive system | No |
| 343 | 6145 value 6 | Increase | 2.31e-05 | BINARY | Illness, injury, bereavement, stress in last 2 years: financial difficulties | No |
| 344 | 22613 value 1 | Increase | 2.37e-05 | BINARY | Worked with paints thinners or glues | No |
| 345 | 41249 value 1002 | Increase | 2.43e-05 | BINARY | Methods of admission to hospital (recoded): elective admission: booked | No |
| 346 | 20003 value 1140861998 | Increase | 2.44e-05 | BINARY | Treatment/medication code: ventolin 100micrograms inhaler | No |
| 347 | 41210 value J439 | Increase | 2.45e-05 | BINARY | Operative procedures - secondary OPCS: Unspecified diagnostic endoscopic retrograde examination of bile duct and pancreatic duct | No |
| 348 | 20514 | Increase | 2.46e-05 | ORDERED | Recent lack of interest or pleasure in doing things | *{Not at all … nearly every day}* |
| 349 | 41200 value J381 | Increase | 2.49e-05 | BINARY | Operative procedures - main OPCS: endoscopic sphincterotomy of sphincter of Oddi and removal of calculus HFQ | No |
| 350 | 20004 value 1484 | Increase | 2.49e-05 | BINARY | Operation code: pilonidal sinus surgery (anal) | No |
| 351 | 41200 value D106 | Increase | 2.51e-05 | BINARY | Operative procedures - main OPCS: revision of mastoidectomy | No |
| 352 | 41204 value M159 | Increase | 2.55e-05 | BINARY | Diagnoses - secondary ICD10: Polyarthrosis, unspecified | No |
| 353 | 100460 | Decrease | 2.71e-05 | ORDERED | Added milk to standard tea | *{No … yes}* |
| 354 | 41202 value E119 | Increase | 2.71e-05 | BINARY | Diagnoses - main ICD10: Non-insulin-dependent diabetes mellitus, without complications | No |
| 355 | 41202 value K429 | Increase | 2.80e-05 | BINARY | Diagnoses - main ICD10: umbilical hernia without obstruction or gangrene | No |
| 356 | 90045 |  | 2.82e-05 | LINEAR | Average acceleration 18:00 - 18:59 | - |
| 357 | 680 (reference=1) | - | 2.92e-05 | MULTINOMIAL | Own or rent accommodation lived in | Own outright |
| 358 | 22506 (reference=114) | - | 2.97e-05 | MULTINOMIAL | Tobacco smoking | Never smoked |
| 359 | 41200 value S472 | Increase | 3.13e-05 | BINARY | Operative procedures - main OPCS: drainage of lesion of skin NEC | No |
| 360 | 6017 | Decrease | 3.20e-05 | ORDERED | Able to walk or cycle unaided for 10 minutes | *{No … yes}* |
| 361 | 41210 value Y534 | Increase | 3.50e-05 | BINARY | Operative procedures - secondary OPCS: Approach to organ under fluoroscopic control | No |
| 362 | 41202 value K801 | Increase | 3.53e-05 | BINARY | Diagnoses - main ICD10: calculus of gallbladder with other cholecystitis | No |
| 363 | 22423 | Increase | 3.66e-05 | LINEAR | LV stroke volume | - |
| 364 | 6164 value 3 | Decrease | 3.70e-05 | BINARY | Types of physical activity in last 4 weeks: Strenuous sports | No |
| 365 | 41200 value T202 | Decrease | 3.70e-05 | BINARY | Operative procedures - main OPCS: Primary repair of inguinal hernia using insert of prosthetic material | No |
| 366 | 2887 | Increase | 3.82e-05 | ORDERED | Number of cigarettes previously smoked daily | *{3 quantity bands}* |
| 367 | 41200 value K633 | Increase | 3.99e-05 | BINARY | Operative procedures - main OPCS: angiocardiography of left side of heart NEC | No |
| 368 | 20003 value 2038459814 | Increase | 4.02e-05 | BINARY | Treatment/medication code: digoxin | No |
| 369 | 41200 value K634 | Increase | 4.09e-05 | BINARY | Operative procedures - main OPCS: Coronary arteriography using two catheters | No |
| 370 | 6154 value 100 | Decrease | 4.10e-05 | BINARY | Medication for pain relief, constipation, heartburn: “None of the above” | “None of the above” option not selected |
| 371 | 41202 value T840 | Increase | 4.11e-05 | BINARY | Diagnoses - main ICD10: mechanical complication of internal joint prosthesis | No |
| 372 | 41204 value G590 | Increase | 4.24e-05 | BINARY | Diagnoses - secondary ICD10: Diabetic mononeuropathy | No |
| 373 | 23304 | Increase | 4.27e-05 | LINEAR | Trunk bone area | - |
| 374 | 90143 | Increase | 4.55e-05 | ORDERED | Fraction acceleration <= 500 milli-gravities | *{3 fraction bands}* |
| 375 | 20002 value 1473 | Increase | 4.61e-05 | BINARY | Non-cancer illness code, self-reported: high cholesterol | No |
| 376 | 41202 value M161 | Increase | 4.82e-05 | BINARY | Diagnoses - main ICD10: other primary coxarthrosis | No |
| 377 | 90145 | Increase | 4.94e-05 | ORDERED | Fraction acceleration <= 700 milli-gravities | *{3 fraction bands}* |
| 378 | 41204 value I251 | Increase | 4.97e-05 | BINARY | Diagnoses - secondary ICD10: Atherosclerotic heart disease | No |
| 379 | 2415 | Increase | 5.06e-05 | BINARY | Had major operations | No |
| 380 | 41204 value R600 | Increase | 5.31e-05 | BINARY | Diagnoses - secondary ICD10: Localised oedema | No |
| 381 | 20107 value 8 | Increase | 5.39e-05 | BINARY | Illnesses of father: high blood pressure | No |
| 382 | 20544 value 12 | Increase | 5.48e-05 | BINARY | Mental health problems ever diagnosed by a professional: Bulimia nervosa | No |
| 383 | 23320 | Increase | 5.54e-05 | LINEAR | Leg BMC (bone mineral content) (left) | - |
| 384 | 41202 value G510 | Increase | 5.70e-05 | BINARY | Diagnoses - main ICD10: Bell's palsy | No |
| 385 | 41202 value I800 | Increase | 5.73e-05 | BINARY | Diagnoses - main ICD10: phlebitis and thrombophlebitis of superficial vessels of lower extremities | No |
| 386 | 23202 | Increase | 6.04e-05 | LINEAR | L1-L4 average width | - |
| 387 | 41204 value M1999 | Increase | 6.09e-05 | BINARY | Diagnoses - secondary ICD10: Arthrosis, unspecified (Site unspecified) | No |
| 388 | 41204 value E059 | Increase | 6.11e-05 | BINARY | Diagnoses - secondary ICD10: Thyrotoxicosis, unspecified | No |
| 389 | 20002 value 1564 | Increase | 6.30e-05 | BINARY | Non-cancer illness code, self-reported: antiphospholipid syndrome | No |
| 390 | 1170 | Increase | 6.62e-05 | ORDERED | Getting up in morning | *{Not at all easy … Very easy}* |
| 391 | 41202 value I251 | Increase | 7.08e-05 | BINARY | Diagnoses - main ICD10: atherosclerotic heart disease | No |
| 392 | 40002 value E149 | Increase | 7.59e-05 | BINARY | Contributory (secondary) causes of death: ICD10: Unspecified diabetes mellitus, without complications | No |
| 393 | 100240 | Increase | 7.82e-05 | BINARY | Coffee consumed | No |
| 394 | 1950 | Increase | 7.83e-05 | BINARY | Sensitivity / hurt feelings | No |
| 395 | 41200 value T315 | Increase | 7.84e-05 | BINARY | Operative procedures - main OPCS: Drainage of anterior abdominal wall | No |
| 396 | 41247 value 2000 | Increase | 8.02e-05 | BINARY | Patient classification on admission (recoded): Day case | No |
| 397 | 41204 value I739 | Increase | 8.16e-05 | BINARY | Diagnoses - secondary ICD10: Peripheral vascular disease, unspecified | No |
| 398 | 2814 | Decrease | 8.21e-05 | BINARY | Ever used hormone-replacement therapy (HRT) | No |
| 399 | 1160 | Decrease | 8.38e-05 | ORDERED | Sleep duration | *{3 duration bands}* |
| 400 | 20002 value 1276 | Increase | 8.39e-05 | BINARY | Non-cancer illness code, self-reported: diabetic eye disease | No |
| 401 | 100580 | Decrease | 8.50e-05 | BINARY | Alcohol consumed | No |
| 402 | 6148 value 100 | Decrease | 8.66e-05 | BINARY | Eye problems/disorders | “None of the above” option not selected |
| 403 | 22421 | Increase | 9.13e-05 | LINEAR | LV end diastolic volume | - |
| 404 | 30050 | Decrease | 1.01e-04 | LINEAR | Mean corpuscular haemoglobin | - |
| 405 | 90141 | Increase | 1.05e-04 | ORDERED | Fraction acceleration <= 450 milli-gravities | *{3 fraction bands}* |
| 406 | 22613 value 0 | Decrease | 1.05e-04 | BINARY | Worked with paints thinners or glues: Rarely/never | No |
| 407 | 20002 value 1625 | Increase | 1.08e-04 | BINARY | Non-cancer illness code, self-reported: cellulitis | No |
| 408 | 41204 value N179 | Increase | 1.08e-04 | BINARY | Diagnoses - secondary ICD10: Acute renal failure, unspecified | No |
| 409 | 90046 | Decrease | 1.09e-04 | LINEAR | Average acceleration 19:00 - 19:59 | - |
| 410 | 41204 value F171 | Increase | 1.12e-04 | BINARY | Diagnoses - secondary ICD10: Mental and behavioural disorders due to use of tobacco, Harmful use | No |
| 411 | 41204 value E109 | Increase | 1.13e-04 | BINARY | Diagnoses - secondary ICD10: Insulin-dependent diabetes mellitus (Without complications) | No |
| 412 | 41204 value Z720 | Increase | 1.14e-04 | BINARY | Diagnoses - secondary ICD10: Tobacco use | No |
| 413 | 41202 value K610 | Increase | 1.16e-04 | BINARY | Diagnoses - main ICD10: |  |
| 414 | 104450 | Increase | 1.19e-04 | ORDERED | Apple intake | *{Half … 4+}* |
| 415 | 41204 value G990 | Increase | 1.19e-04 | BINARY | Diagnoses - secondary ICD10: Autonomic neuropathy in endocrine and metabolic diseases | No |
| 416 | 90142 | Decrease | 1.21e-04 | ORDERED | Fraction acceleration <= 475 milli-gravities | *{3 fraction bands}* |
| 417 | 3637 | Increase | 1.22e-04 | ORDERED | Frequency of other exercises in last 4 weeks | *{Once in the last 4 weeks … every day}* |
| 418 | 20002 value 1594 | Increase | 1.23e-04 | BINARY | Non-cancer illness code, self-reported: respiratory infection | No |
| 419 | 5119 | Decrease | 1.23e-04 | LINEAR | 3mm cylindrical power (left) | - |
| 420 | 41204 value J849 | Increase | 1.24e-04 | BINARY | Diagnoses - secondary ICD10: Interstitial pulmonary disease, unspecified | No |
| 421 | 20003 value 1141174508 | Increase | 1.24e-04 | BINARY | Treatment/medication code: reductil 10mg capsule | No |
| 422 | 41202 value K409 | Decrease | 1.25e-04 | BINARY | Diagnoses - main ICD10: Unilateral or unspecified inguinal hernia, without obstruction or gangrene | No |
| 423 | 41203 value 0093 | Decrease | 1.26e-04 | BINARY | Diagnoses - main ICD9: Diarrhoea, of presumed infectious origin | No |
| 424 | 41210 value Y508 | Increase | 1.26e-04 | BINARY | Operative procedures - secondary OPCS: Other specified approach through abdominal cavity | No |
| 425 | 41204 value M109 | Increase | 1.26e-04 | BINARY | Diagnoses - secondary ICD10: Gout, unspecified | No |
| 426 | 3606 | Increase | 1.29e-04 | BINARY | Chest pain or discomfort walking normally | No |
| 427 | 41230 value 5PJ | Increase | 1.31e-04 | BINARY | PCT where patients GP was registered: STOKE ON TRENT PCT | No |
| 428 | 981 | Decrease | 1.34e-04 | ORDERED | Duration walking for pleasure | *{Less than 15 minutes … between 2 and 3 hours}* |
| 429 | 25402 | Increase | 1.36e-04 | LINEAR | Mean OD in inferior cerebellar peduncle on FA skeleton (right) | - |
| 430 | 6145 value 1 | Increase | 1.37e-04 | BINARY | Illness, injury, bereavement, stress in last 2 years: serious illness, injury or assault to yourself | No |
| 431 | 2966 | Decrease | 1.40e-04 | LINEAR | Age high blood pressure diagnosed | - |
| 432 | 1883 | Increase | 1.41e-04 | ORDERED | Number of full sisters | *{0 … 10}* |
| 433 | 41204 value E662 | Increase | 1.42e-04 | BINARY | Diagnoses - secondary ICD10: Extreme obesity with alveolar hypoventilation | No |
| 434 | 41229 value 5PJ | Increase | 1.42e-04 | BINARY | PCT responsible for patient data: STOKE ON TRENT PCT | No |
| 435 | 2159 | Increase | 1.47e-04 | BINARY | Ever had same-sex intercourse | No |
| 436 | 41204 value N40 | Increase | 1.51e-04 | BINARY | Diagnoses - secondary ICD10: Hyperplasia of prostate | No |
| 437 | 90013 | Decrease | 1.53e-04 | LINEAR | Standard deviation of acceleration | - |
| 438 | 1960 | Increase | 1.54e-04 | BINARY | Fed-up feelings | No |
| 439 | 6333 | Decrease | 1.56e-04 | LINEAR | Duration spent answering each puzzle | - |
| 440 | 22407 | Increase | 1.62e-04 | LINEAR | Visceral adipose tissue volume (VAT) | - |
| 441 | 41200 value W852 | Increase | 1.64e-04 | BINARY | Operative procedures - main OPCS: endoscopic irrigation of knee joint | No |
| 442 | 1279 | Increase | 1.73e-04 | ORDERED | Exposure to tobacco smoke outside home | *{3 time bands}* |
| 443 | 41249 value 2003 | Increase | 1.75e-04 | BINARY | Methods of admission to hospital (recoded): emergency admission: bed bureau | No |
| 444 | 90088 | Decrease | 1.91e-04 | LINEAR | No-wear time bias adjusted acceleration standard deviation | - |
| 445 | 20004 value 1368 | Decrease | 1.95e-04 | BINARY | Operation code: mastectomy | No |
| 446 | 41210 value M763 | Increase | 2.07e-04 | BINARY | Operative procedures - secondary OPCS: Optical urethrotomy | No |
| 447 | 1269 | Increase | 2.13e-04 | ORDERED | Exposure to tobacco smoke at home | *{3 quantity bands}* |
| 448 | 41200 value L746 | Increase | 2.22e-04 | BINARY | Operative procedures - main OPCS: Creation of graft fistula for dialysis | No |
| 449 | 41204 value I501 | Increase | 2.23e-04 | BINARY | Diagnoses - secondary ICD10: Left ventricular failure | No |
| 450 | 41204 value I872 | Increase | 2.34e-04 | BINARY | Diagnoses - secondary ICD10: Venous insufficiency (chronic) (peripheral) | No |
| 451 | 90146 | Increase | 2.36e-04 | ORDERED | Fraction acceleration <= 800 milli-gravities | *{3 fraction bands}* |
| 452 | 806 | Increase | 2.45e-04 | ORDERED | Job involves mainly walking or standing | *{Never/rarely … always}* |
| 453 | 25162 | Decrease | 2.47e-04 | LINEAR | Mean MO in inferior cerebellar peduncle on FA skeleton (right) | - |
| 454 | 41202 value E113 | Increase | 2.52e-04 | BINARY | Diagnoses - main ICD10: with ophthalmic complications | No |
| 455 | 41202 value E115 | Increase | 2.61e-04 | BINARY | Diagnoses - main ICD10: With peripheral circulatory complications | No |
| 456 | 20004 value 1404 | Increase | 2.61e-04 | BINARY | Operation code: umbilical hernia repair | No |
| 457 | 6153 value 4 | Decrease | 2.63e-04 | BINARY | Medication for cholesterol, blood pressure, diabetes, or take exogenous hormones: hormone replacement therapy | No |
| 458 | 41210 value S063 | Increase | 2.67e-04 | BINARY | Operative procedures - secondary OPCS: Shave excision of lesion of skin of head or neck | No |
| 459 | 41202 value I48 | Increase | 2.71e-04 | BINARY | Diagnoses - main ICD10: Atrial fibrillation and flutter | No |
| 460 | 41202 value E272 | Increase | 2.77e-04 | BINARY | Diagnoses - main ICD10: Addisonian crisis | No |
| 461 | 21004 | Increase | 2.78e-04 | ORDERED | Number of puzzles correct | *{1 … 18}* |
| 462 | 41246 value 1800 | Increase | 2.79e-04 | BINARY | Treatment speciality of consultant (recoded): nephrology | No |
| 463 | 90091 | Decrease | 2.85e-04 | LINEAR | No-wear time bias adjusted acceleration maximum | - |
| 464 | 41210 value E852 | Increase | 3.00e-04 | BINARY | Operative procedures - secondary OPCS: Non-invasive ventilation NEC | No |
| 465 | 41201 value V814 | Increase | 3.09e-04 | BINARY | External causes: Person injured while boarding or alighting from railway train or railway vehicle | No |
| 466 | 6382 | Increase | 3.14e-04 | ORDERED | Number of puzzles correct | {0 … 18} |
| 467 | 41210 value Y731 | Increase | 3.23e-04 | BINARY | Operative procedures - secondary OPCS: Cardiopulmonary bypass | No |
| 468 | 90140 | Increase | 3.27e-04 | ORDERED | Fraction acceleration <= 425 milli-gravities | *{3 fraction bands}* |
| 469 | 3085 | Increase | 3.29e-04 | LINEAR | Heel Broadband ultrasound attenuation (BUA), manual entry | - |
| 470 | 41210 value W852 | Increase | 3.47e-04 | BINARY | Operative procedures - secondary OPCS: Endoscopic irrigation of knee joint | No |
| 471 | 41200 value E353 | Decrease | 3.50e-04 | BINARY | Operative procedures - main OPCS: Endoscopic destruction of lesion of larynx | No |
| 472 | 41229 value 5PK | Increase | 3.56e-04 | BINARY | PCT responsible for patient data: SOUTH STAFFORDSHIRE PCT | No |
| 473 | 41202 value K297 | Increase | 3.62e-04 | BINARY | Diagnoses - main ICD10: gastritis, unspecified | No |
| 474 | 104130 | Increase | 3.67e-04 | ORDERED | Beetroot intake | *{quarter … 3+}* |
| 475 | 41210 value W784 | Increase | 3.68e-04 | BINARY | Operative procedures - secondary OPCS: Limited release of contracture of capsule of joint | No |
| 476 | 4194 | Decrease | 3.71e-04 | LINEAR | Pulse rate | - |
| 477 | 90138 | Increase | 3.73e-04 | ORDERED | Fraction acceleration <= 375 milli-gravities | *{3 fraction bands}* |
| 478 | 23228 | Increase | 3.78e-04 | LINEAR | Leg BMC (bone mineral content) (right) | - |
| 479 | 41230 value 5PK | Increase | 3.99e-04 | BINARY | PCT where patients GP was registered: SOUTH STAFFORDSHIRE PCT | No |
| 480 | 41210 value D162 | Increase | 4.07e-04 | BINARY | Operative procedures - secondary OPCS: Graft replacement of ossicular chain | No |
| 481 | 41204 value G632 | Increase | 4.23e-04 | BINARY | Diagnoses - secondary ICD10: Diabetic polyneuropathy | No |
| 482 | 41210 value H289 | Increase | 4.28e-04 | BINARY | Operative procedures - secondary OPCS: Unspecified diagnostic endoscopic examination of sigmoid colon using rigid sigmoidoscope | No |
| 483 | 20003 value 1141152590 | Increase | 4.30e-04 | BINARY | Treatment/medication code: glimepiride | No |
| 484 | 23315 | Increase | 4.36e-04 | LINEAR | Leg bone area (left) | - |
| 485 | 51 |  | 4.44e-04 | LINEAR | Seated height | - |
| 486 | 6019 (reference=1) | - | 4.46e-04 | MULTINOMIAL | ECG/bike method for fitness test | Bicycle |
| 487 | 20003 value 1141188442 | Increase | 4.53e-04 | BINARY | Treatment/medication code: glucosamine product | No |
| 488 | 41210 value Z275 | Increase | 4.59e-04 | BINARY | Operative procedures - secondary OPCS: Jejunum | No |
| 489 | 20519 | Increase | 4.68e-04 | ORDERED | Recent feelings of tiredness or low energy | *{Not at all … nearly every day}* |
| 490 | 41210 value S577 | Increase | 4.68e-04 | BINARY | Operative procedures - secondary OPCS: Dressing of skin using vacuum assisted closure device NEC | No |
| 491 | 100022 | Decrease | 4.73e-04 | ORDERED | Alcohol | *{3 intake bands}* |
| 492 | 20003 value 1141168574 | Decrease | 4.79e-04 | BINARY | Treatment/medication code: raloxifene hydrochloride | No |
| 493 | 5118 | Decrease | 4.87e-04 | LINEAR | 6mm cylindrical power (left) | - |
| 494 | 41203 value 4785 | Decrease | 4.89e-04 | BINARY | Diagnoses - main ICD9: Other diseases of vocal cords | No |
| 495 | 41246 value 1170 | Increase | 4.91e-04 | BINARY | Treatment speciality of consultant (recoded): cardiothoracic surgery | No |
| 496 | 41204 value R060 | Increase | 4.93e-04 | BINARY | Diagnoses - secondary ICD10: Dyspnoea | No |
| 497 | 2060 | Increase | 5.00e-04 | ORDERED | Frequency of unenthusiasm / disinterest in last 2 weeks | {Not at all … nearly every day} |
| 498 | 90129 | Increase | 5.10e-04 | LINEAR | Fraction acceleration <= 150 milli-gravities | - |
| 499 | 22501 | Decrease | 5.21e-04 | LINEAR | Year ended full time education | *-* |
| 500 | 20004 value 1586 | Decrease | 5.25e-04 | BINARY | Operation code: varicocoele / varicocele surgery | No |
| 501 | 41200 value O172 | Decrease | 5.36e-04 | BINARY | Operative procedures - main OPCS: Remanipulation of fracture of long bone and rigid internal fixation NEC | No |
| 502 | 20003 value 1140888646 | Increase | 5.36e-04 | BINARY | Treatment/medication code: felodipine | No |
| 503 | 728 | Decrease | 5.75e-04 | ORDERED | Number of vehicles in household | *{0 … 4+}* |
| 504 | 20049 value 433 | Increase | 5.77e-04 | BINARY | Blood sample #, note contents: poor venous access /flow | No |
| 505 | 20002 value 1112 | Increase | 5.86e-04 | BINARY | Non-cancer illness code, self-reported: chronic obstructive airways disease/copd | No |
| 506 | 41204 value M199 | Increase | 5.88e-04 | BINARY | Diagnoses - secondary ICD10: M19.9 Arthrosis, unspecified | No |
| 507 | 1120 | Increase | 5.91e-04 | ORDERED | Weekly usage of mobile phone in last 3 months | *{*< *5min … >6 hour}* |
| 508 | 41204 value I978 | Decrease | 5.95e-04 | BINARY | Diagnoses - secondary ICD10: Other postprocedural disorders of circulatory system, not elsewhere classified | No |
| 509 | 20003 value 1140866280 | Increase | 6.07e-04 | BINARY | Treatment/medication code: bumetanide | No |
| 510 | 22601 value 41413308 | Increase | 6.14e-04 | BINARY | Job coding: telephonist telephone/switchboard operator | No |
| 511 | 22617 value 4141 | Increase | 6.14e-04 | BINARY | Job SOC coding: Telephonists | No |
| 512 | 41210 value Z675 | Increase | 6.18e-04 | BINARY | Operative procedures - secondary OPCS: Lumbar intervertebral joint | No |
| 513 | 77 | Increase | 6.32e-04 | LINEAR | Heel bone ultrasound T-score, manual entry | - |
| 514 | 20003 value 99999 | Increase | 6.57e-04 | BINARY | Treatment/medication code: free-text entry, unable to be coded | No |
| 515 | 20110 value 1 | Increase | 6.64e-04 | BINARY | Illnesses of mother: heart disease | No |
| 516 | 90130 | Increase | 6.65e-04 | LINEAR | Fraction acceleration <= 175 milli-gravities | - |
| 517 | 41200 value H582 | Increase | 6.72e-04 | BINARY | Operative procedures - main OPCS: Drainage of perianal abscess | No |
| 518 | 30130 | Increase | 6.74e-04 | LINEAR | Monocyte count | - |
| 519 | 20002 value 1459 | Increase | 6.77e-04 | BINARY | Non-cancer illness code, self-reported: colitis/not crohns or ulcerative colitis | No |
| 520 | 25034 | Decrease | 6.84e-04 | LINEAR | Median T2star in hippocampus (left) | - |
| 521 | 23316 | Increase | 6.91e-04 | LINEAR | Leg bone area (right) | - |
| 522 | 41210 value J411 | Increase | 7.08e-04 | BINARY | Operative procedures - secondary OPCS: Endoscopic retrograde extraction of calculus from bile duct | No |
| 523 | 41204 value R02 | Increase | 7.09e-04 | BINARY | Diagnoses - secondary ICD10: Gangrene, not elsewhere classified | No |
| 524 | 41204 value Z950 | Increase | 7.15e-04 | BINARY | Diagnoses - secondary ICD10: Presence of cardiac pacemaker | No |
| 525 | 1190 |  | 7.44e-04 |  |  |  |
| 526 | 41204 value F329 | Increase | 7.51e-04 | BINARY | Diagnoses - secondary ICD10: Depressive episode, unspecified | No |
| 527 | 41204 value K828 | Increase | 7.53e-04 | BINARY | Diagnoses - secondary ICD10: Other specified diseases of gallbladder | No |
| 528 | 1488 | Decrease | 7.64e-04 | LINEAR | Tea intake | - |
| 529 | 41204 value R001 | Increase | 7.64e-04 | BINARY | Diagnoses - secondary ICD10: Bradycardia, unspecified | No |
| 530 | 20507 | Increase | 7.74e-04 | ORDERED | Recent feelings of inadequacy | *{Not at all … Nearly every day}* |
| 531 | 41210 value K453 | Increase | 7.75e-04 | BINARY | Operative procedures - secondary OPCS: Anastomosis of mammary artery to left anterior descending coronary artery | No |
| 532 | 41202 value G479 | Increase | 7.76e-04 | BINARY | Diagnoses - main ICD10: sleep disorder, unspecified | No |
| 533 | 30040 | Decrease | 7.77e-04 | LINEAR | Mean corpuscular volume | - |
| 534 | 90128 | Increase | 7.78e-04 | LINEAR | Fraction acceleration <= 125 milli-gravities | - |
| 535 | 94 | Increase | 7.84e-04 | LINEAR | Diastolic blood pressure, manual reading | - |
| 536 | 41230 value 5LL | Increase | 7.87e-04 | BINARY | PCT where patients GP was registered | ASHFORD PCT |
| 537 | 41200 value W822 | Increase | 7.88e-04 | BINARY | Operative procedures - main OPCS: Endoscopic resection of semilunar cartilage NEC | No |
| 538 | 5507 | Decrease | 7.92e-04 | ORDERED | Leg pain on walking: action taken | *{Stop … continue at same pace}* |
| 539 | 41202 value R072 | Increase | 7.97e-04 | BINARY | Diagnoses - main ICD10: Precordial pain | No |
| 540 | 20002 value 1350 | Increase | 8.04e-04 | BINARY | Non-cancer illness code, self-reported: polycystic ovaries/polycystic ovarian syndrome | No |
| 541 | 25734 | Increase | 8.06e-04 | LINEAR | Inverted signal-to-noise ratio in T1 | - |
| 542 | 41229 value 5PQ | Decrease | 8.11e-04 | BINARY | PCT responsible for patient data: NORFOLK PCT | No |
| 543 | 41230 value 5PQ | Decrease | 8.11e-04 | BINARY | PCT where patients GP was registered: NORFOLK PCT | No |
| 544 | 41246 value 1430 | Increase | 8.21e-04 | BINARY | Treatment speciality of consultant (recoded): ear, nose and throat | No |
| 545 | 6159 value 8 | Increase | 8.51e-04 | BINARY | Pain type(s) experienced in last month: pain all over the body | No |
| 546 | 5237 | Increase | 8.67e-04 | ORDERED | 3mm index of best keratometry results (right) | *{Numeric bands}* |
| 547 | 41204 value I849 | Increase | 8.71e-04 | BINARY | Diagnoses - secondary ICD10: Unspecified haemorrhoids without complication | No |
| 548 | 41204 value V814 | Increase | 8.73e-04 | BINARY | Diagnoses - secondary ICD10: Person injured while boarding or alighting from railway train or railway vehicle | No |
| 549 | 41200 value J439 | Increase | 8.92e-04 | BINARY | Unspecified diagnostic endoscopic retrograde examination of bile duct and pancreatic duct | No |
| 550 | 41202 value E109 | Increase | 9.01e-04 | BINARY | Diagnoses - main ICD10: Insulin-dependent diabetes mellitus, without complications | No |
| 551 | 41210 value Y714 | Increase | 9.22e-04 |  | Operative procedures - secondary OPCS: Failed minimal access approach converted to open | No |
| 552 | 41210 value Z705 | Decrease | 9.24e-04 | BINARY | Operative procedures - secondary OPCS: Lower end of radius NEC | No |
| 553 | 23309 | Increase | 9.37e-04 | LINEAR | Ribs bone area | - |
| 554 | 41201 value X519 | Increase | 9.40e-04 | BINARY | External causes: Unspecified place | No |
| 555 | 41202 value M0690 | Increase | 9.48e-04 | BINARY | Diagnoses - main ICD10: Rheumatoid arthritis, unspecified (Multiple sites) | No |
| 556 | 41200 value G151 | Increase | 9.57e-04 | BINARY | Operative procedures - main OPCS: Fibreoptic endoscopic removal of foreign body from oesophagus | No |
| 557 | 41245 value 1080 | Increase | 9.58e-04 | BINARY | Main speciality of consultant (recoded): cardiothoracic surgery | No |
| 558 | 41210 value C712 | Increase | 9.62e-04 | BINARY | Operative procedures - secondary OPCS: Phacoemulsification of lens | No |
| 559 | 20004 value 1448 | Increase | 9.63e-04 | BINARY | Operation code: stomach surgery | No |
| 560 | 41202 value I839 | Increase | 9.64e-04 | BINARY | Diagnoses - main ICD10: varicose veins of lower extremities without ulcer or inflammation | No |
| 561 | 41200 value M814 | Increase | 9.85e-04 | BINARY | Dilation of meatus of urethra | No |
| 562 | 41204 value Y534 | Increase | 9.94e-04 | BINARY | Diagnoses - secondary ICD10: Other laxatives | No |
| 563 | 22507 | Increase | 1.00e-03 | LINEAR | Age of stopping smoking | - |
| 564 | 41204 value S060 | Increase | 1.02e-03 | BINARY | Diagnoses - secondary ICD10: Concussion | No |
| 565 | 20003 value 1140879902 | Increase | 1.03e-03 | BINARY | Treatment/medication code: coal tar product | No |
| 566 | 1339 | Increase | 1.03e-03 | ORDERED-LOGISTIC | Non-oily fish intake | {Never … Once or more daily} |
| 567 | 20003 value 1141152228 | Increase | 1.06e-03 | BINARY | Treatment/medication code: elleste-solo mx 40 patch | No |
| 568 | 41202 value M2332 | Increase | 1.06e-03 | BINARY | Diagnoses - main ICD10: Other meniscus derangements | No |
| 569 | 4825 | Increase | 1.06e-03 | ORDERED | Noisy workplace | {No .. yes, for more than 5 years} |
| 570 | 6142 value 3 | Decrease | 1.06e-03 | BINARY | Current employment status: looking after home and/or family | No |
| 571 | 41200 value E852 | Increase | 1.06e-03 | BINARY | Operative procedures - main OPCS: Non-invasive ventilation NEC | No |
| 572 | 41204 value K660 | Increase | 1.08e-03 | BINARY | Diagnoses - secondary ICD10: Peritoneal adhesions | No |
| 573 | 22601 value 81492535 | Increase | 1.09e-03 | BINARY | Job coding: building site foreman or supervisor | No |
| 574 | 20003 value 1140873548 | Decrease | 1.13e-03 | BINARY | Treatment/medication code: oxytetracycline | No |
| 575 | 41204 value I469 | Increase | 1.14e-03 | BINARY | Diagnoses - secondary ICD10: Cardiac arrest, unspecified | No |
| 576 | 3456 | Increase | 1.15e-03 | ORDERED-LOGISTIC | Number of cigarettes currently smoked daily (current cigarette smokers) | {3 quantity bands} |
| 577 | 30010 | Increase | 1.15e-03 | LINEAR | Red blood cell (erythrocyte) count | - |
| 578 | 41202 value H269 | Increase | 1.16e-03 | BINARY | Diagnoses - main ICD10: Cataract, unspecified | No |
| 579 | 6145 value 100 | Decrease | 1.18e-03 | BINARY | Illness, injury, bereavement, stress in last 2 years: None | Not None |
| 580 | 41204 value W188 | Decrease | 1.21e-03 | BINARY | Diagnoses - secondary ICD10: W18.8 Other specified place | No |
| 581 | 20002 value 1464 | Increase | 1.22e-03 | BINARY | Non-cancer illness code self-reported: rheumatoid arthritis | No |
| 582 | 20110 value 10 | Decrease | 1.22e-03 | BINARY | Illnesses of mother: Alzheimer's disease/dementia | No |
| 583 | 23310 | Increase | 1.23e-03 | LINEAR | Ribs BMC (bone mineral content) | - |
| 584 | 41210 value Z787 | Increase | 1.23e-03 | BINARY | Operative procedures - secondary OPCS: Z78.7 Patella | No |
| 585 | 2986 | Decrease | 1.25e-03 | BINARY | Started insulin within one year diagnosis of diabetes | No |
| 586 | 20087 value 159 | Decrease | 1.26e-03 | BINARY | Types of spread used on bread/crackers: Butter on bread/crackers | No |
| 587 | 6162 value 4 | Decrease | 1.26e-03 | BINARY | Types of transport used (excluding work): Cycle | No |

Results of main analysis, adjusting for age, sex and the first 10 genetic principal components.

^1^ Direction of change of outcome with genetic predisposition to higher BMI.

^2^ For multinomial logistic regression results a single P value was calculated for each model as a whole, using the likelihood ratio chi-square test. ^1^ In R the smallest possible number is 2.23e-308 (specified in .Machine$double.xmin).

^3^ Information on categories for multinomial, ordinal and binary regression results: reference category for multinomial regression results, baseline category for binary logistic regression results, and category ordering for ordered logistic regression results. For example, “*{thinner … plumper}*” for field 1687 means that there are categories ranging from thinner to plumper where thinner is coded with the smallest value and plumper with the largest value.

^4^ In addition to the field ID, this column also contains the reference value for multinomial regression results, and the field value for which a binary variable was generated for categorical (multiple) fields.

Supplementary table E: Results of follow-up analysis of the associations between nervousness / worrying traits (outcomes) and BMI (exposure)

|  | **Nervous person (FID=1970)** | **Being a worrier (FID=1980)** | **Tense / highly strung (FID=1990)** | **Suffering from nerves (FID=2010)** |
| --- | --- | --- | --- | --- |
| N | 325,552 | 325,523 | 324,146 | 322,236 |
| **OBSERVATIONAL ESTIMATES ^7^** | | | | |
| Observational association ^3^ * | 0.964 [0.962, 0.966] | 0.981 [0.980, 0.983] | 0.983 [0.981, 0.985] | 0.993 [0.991, 0.994] |
| **CAUSAL ESTIMATES** | | | | |
| **Estimates using two-stage IV probit regression** ^8^ | | | | |
| Effect estimate with 97 SNP score ^1^ | -0.039 [-0.046, -0.031] | -0.017 [-0.024, -0.010] | -0.028 [-0.036, -0.020] | -0.024 [-0.031, -0.016] |
| Effect estimate with 97 SNP score – approximate change of odds ^4^ * | 0.940 [0.929, 0.951] | 0.973 [0.963, 0.984] | 0.957 [0.945, 0.969] | 0.963 [0.951, 0.974] |
| Effect estimate with 97 SNP score, in terms of SD change of BMI ^11^ | -0.184 [-0.219, -0.149] | -0.081 [-0.113, -0.048] | -0.131 [-0.169, -0.093] | -0.113 [-0.149, -0.077] |
| Effect estimate with 97 SNP score, in terms of SD change of BMI, approximate change of log odds ^6 +^ | -0.294 [-0.350, -0.238] | -0.129 [-0.181, -0.077] | -0.210 [-0.270, -0.149] | -0.181 [-0.239, -0.123] |
| Effect estimate with 96 SNP score (excluding FTO SNP) ^1^ | -0.040 [-0.048, -0.032] | -0.021 [-0.029, -0.014] | -0.031 [-0.040, -0.022] | -0.026 [-0.034, -0.018] |
| Effect estimate with 96 SNP score (excluding FTO SNP), approximate change of odds ^4^ * | 0.938 [0.926, 0.950] | 0.967 [0.955, 0.978] | 0.952 [0.939, 0.965] | 0.959 [0.946, 0.972] |
| Effect estimate with FTO SNP ^1^ | -0.031 [-0.050, -0.012] | 0.012 [-0.006, 0.029] | -0.004 [-0.025, 0.017] | -0.008 [-0.027, 0.012] |
| Effect estimate with FTO SNP, approximate change of odds ^4^ * | 0.951 [0.922, 0.981] | 1.019 [0.991, 1.048] | 0.993 [0.961, 1.027] | 0.988 [0.957, 1.020] |
| **Estimates using MR-Egger ^2^** | | | | |
| Causal effect estimate ^+^ | -0.189 [-0.411, 0.033] | -0.048 [-0.237, 0.141] | -0.132 [-0.373, 0.109] | -0.176 [-0.405, 0.053] |
| Intercept estimate | -0.002 [-0.008, 0.004] | -0.002 [-0.007, 0.004] | -0.002 [-0.008, 0.005] | 0.001 [-0.006, 0.007] |
| $I_{GX}^{2}$statistic | 0.892 | | | |
| Bias adjusted causal effect estimate ^5 +^ | -0.214 [-0.459, 0.031] | -0.055 [-0.267, 0.157] | -0.145 [-0.416, 0.126] | -0.198 [-0.453, 0.058] |
| **Estimates using weighted median ^2^** | | | | |
| Weighted median effect estimate ^+^ | -0.219 [-0.314, -0.125] | -0.084 [-0.162, -0.007] | -0.171 [-0.269, -0.074] | -0.079 [-0.172, 0.015] |
| **Estimates using mode based estimate ^2^** | | | | |
| Simple, *ϕ=0.75* ^+^ | -0.079 [-0.312, 0.155] | -0.161 [-0.453, 0.131] | -0.272 [-0.590, 0.047] | -0.024 [-0.314, 0.266] |
| Weighted, *ϕ=0.75* ^+^ | -0.159 [-0.294, -0.025] | 0.063 [-0.054, 0.180] | -0.119 [-0.279, 0.041] | -0.048 [-0.185, 0.089] |
| **DISCOVERY AND REPLICATION ESTIMATES ^9, 10^** | | | | |
| Discovery effect estimate with 97 SNP score ^1^ | -0.037 [-0.049, -0.025] | -0.017, [-0.028, -0.005] | -0.033 [-0.046, -0.019] | -0.026 [-0.039, -0.013] |
| Discovery Effect estimate with 97 SNP score – approximate change of odds ^4^ * | 0.943 [0.924, 0.962] | 0.973 [0.956, 0.991] | 0.949 [0.929, 0.969] | 0.960 [0.940, 0.980] |
| Replication effect estimate with 97 SNP score ^1^ | -0.039 [-0.048, -0.030] | -0.014 [-0.022, -0.005] | -0.023 [-0.033, -0.013] | -0.023 [-0.032, -0.014] |
| Replication Effect estimate with 97 SNP score – approximate change of odds ^4^ * | 0.939 [0.926, 0.953] | 0.978 [0.965, 0.991] | 0.964 [0.949, 0.979] | 0.964 [0.950, 0.979] |

BMI: body mass index; FID: field identifier; SD: standard deviation; IV: instrumental variable.

^1^ Coefficient [95% confidence interval] from instrument variable probit models (ivprobit Stata command), for a 1 kg/m^2^ higher BMI.

^2^ Two sample analyses use the SNP-BMI associations from Locke GWAS (33), and SNP-outcome associations estimated in UK Biobank (adjusted for and first 10 genetic principal components).

^3^ Estimate of the odds ratio of outcome for a 1 kg/m^2^ increase in BMI.

^4^ Estimate of the odds ratio of outcome for a 1 kg/m^2^ increase in BMI, calculated by taking the exponent of 1.6 times the probit estimate (35).

^5^ Bias adjusted causal effect estimate using MR-Egger with SIMEX.

^6^ Effect estimate for SD change in BMI and logistic approximation (calculated as 1.6 times the probit estimate), for comparison with two-sample approaches, as these use the BMI standardised SNP associations from the GIANT GWAS (33).

^7^ Adjusted for age and sex.

^8^ Adjusted for age, sex and first 10 genetic principal components (see Table F in S1 Text for results of sensitivity analysis, adjusting for age, sex and first 40 genetic principal components).

^9^ Discovery results are estimated on the UK Biobank sample used in the PHESANT application note usage example (30).

^10^ Sample size for discovery samples: Field 1970: 111,746; Field 1980: 111,673; Field 1990: 111,161; Field 2010: 110,451.

Sample size for replication samples: Field 1970: 216,311; Field 1980: 216,301; Field 1990: 215,435; Field 2010: 214,152.

^11^ Coefficient [95% confidence interval] from instrument variable probit models (ivprobit Stata command), for a 1 SD higher BMI.

^+^ Estimates are in terms of change of log odds of outcome variable for a 1 SD higher BMI.

* Estimates are in terms of odds ratio of outcome variable for a 1 kg/m^2^ higher BMI.

Supplementary table F: Results of follow-up analysis of nervousness / worrying traits using IV probit regression – main analysis and sensitivity analysis

|  | **Nervous person (1970)** | **Being a worrier (1980)** | **Tense / highly strung (1990)** | **Suffering from nerves (2010)** |
| --- | --- | --- | --- | --- |
| N | 325,552 | 325,523 | 324,146 | 322,236 |
| Effect estimate with 97 SNP score ^1^ | -0.039 [-0.046, -0.031] | -0.017 [-0.024, -0.010] | -0.028 [-0.036, -0.020] | -0.024 [-0.031, -0.016] |
| *Sensitivity* | *-0.039 [-0.046, -0.031]* | *-0.017 [-0.024, -0.010]* | *-0.028 [-0.036, -0.020]* | *-0.024 [-0.032, -0.016]* |
| Effect estimate with 96 SNP score (excluding FTO SNP) ^1^ | -0.040 [-0.048, -0.032] | -0.021 [-0.029, -0.014] | -0.031 [-0.040, -0.022] | -0.026 [-0.034, -0.018] |
| *Sensitivity* | *-0.040 [-0.048, -0.032]* | *-0.021 [-0.029, -0.014]* | *-0.032 [-0.040, -0.023]* | *-0.027 [-0.035, -0.018]* |
| Effect estimate with FTO SNP ^1^ | -0.031 [-0.050, -0.012] | 0.012 [-0.006, 0.029] | -0.004 [-0.025, 0.017] | -0.008 [-0.027, 0.012] |
| *Sensitivity* | *-0.031 [-0.050, -0.011]* | *0.012 [-0.006, 0.029]* | *-0.004 [-0.024, 0.017]* | *-0.007 [-0.027, 0.013]* |

This table contains results shown in “Estimates using two-stage IV probit regression” section of Supplementary table E, and results of sensitivity analyses.

Main analyses: Adjusted for age, sex and first 10 genetic principal components. Sensitivity analyses: Adjusted for age, sex and first 40 genetic principal components

^1^ Coefficient [95% confidence interval] from instrument variable probit models (ivprobit Stata command), for a 1 kg/m^2^ higher BMI.

Supplementary table G: Contingency tables for nervousness phenotypes

a) “Nervous person” (FID=1970) versus “Being a worrier” (FID=1980)

|  | **1980** | |
| --- | --- | --- |
| **1970** | No | Yes |
| No | 42.12 | 33.91 |
| Yes | 1.76 | 22.22 |

Accuracy = 64.33%

b) “Nervous person” (FID=1970) versus “Tense/highly strung” (FID=1990)

|  | **1990** | |
| --- | --- | --- |
| **1970** | No | Yes |
| No | 70.73 | 6.20 |
| Yes | 12.28 | 10.79 |

Accuracy = 81.52%

c) “Nervous person” (FID=1970) versus “Suffering from nerves” (FID=2010)

|  | **2010** | |
| --- | --- | --- |
| **1970** | No | Yes |
| No | 69.72 | 7.40 |
| Yes | 9.11 | 13.77 |

Accuracy = 83.49%

d) “Being a worrier” (FID=1980) versus “Tense/highly strung” (FID=1990)

|  | **1990** | |
| --- | --- | --- |
| **1980** | No | Yes |
| No | 42.09 | 1.85 |
| Yes | 40.57 | 15.50 |

Accuracy = 57.59%

e) “Being a worrier” (FID=1980) versus “Suffering from nerves” (FID=2010)

|  | **2010** | |
| --- | --- | --- |
| **1980** | No | Yes |
| No | 40.93 | 3.18 |
| Yes | 37.51 | 18.38 |

Accuracy = 59.31%

f) “Tense/highly strung” (FID=1990) versus “Suffering from nerves” (FID=2010)

|  | **2010** | |
| --- | --- | --- |
| **1990** | No | Yes |
| No | 71.78 | 11.59 |
| Yes | 7.31 | 9.33 |

Accuracy = 81.11%

FID: Field identifier.

Contingency tables are given in terms of the percentage of participants in each group.

Accuracy reflects the proportion of agreement between the values of two traits.

Supplementary table H: Results where association differs between main analysis and sensitivity analysis (additionally adjusting for assessment centre and genetic batch)

| Field ID | P value (main analysis) | P value (sensitivity) ^1^ | Numbers in categories |
| --- | --- | --- | --- |
| 20085 value 3 | 0.068 | <2.23e-308 | 47264/255 |
| 20087 value 177 | 0.891 | <2.23e-308 | 47475/44 |
| 20087 value 186 | 0.231 | <2.23e-308 | 47468/51 |
| 20087 value 192 | 0.729 | <2.23e-308 | 47423/96 |
| 20087 value 194 | 0.683 | <2.23e-308 | 47435/84 |
| 20087 value 195 | 0.133 | <2.23e-308 | 47388/131 |
| 20090 value 369 | 0.422 | <2.23e-308 | 47313/206 |
| 20090 value 378 | 0.730 | <2.23e-308 | 47486/33 |
| 20090 value 381 | 0.991 | <2.23e-308 | 47499/20 |
| 20090 value 382 | 0.319 | <2.23e-308 | 47454/65 |
| 20090 value 387 | 0.182 | <2.23e-308 | 47492/27 |
| 20090 value 390 | 0.339 | <2.23e-308 | 47495/24 |
| 20090 value 393 | 0.934 | <2.23e-308 | 47490/29 |
| 20097 value 3 | 0.575 | 4.40e-16 | 47391/128 |
| 20101 value 3 | 0.274 | <2.23e-308 | 47351/168 |
| 20103 value 1 | 0.418 | <2.23e-308 | 47393/126 |
| 20104 value 1 | 0.257 | <2.23e-308 | 47196/323 |
| 20104 value 3 | 0.841 | <2.23e-308 | 47460/59 |
| 20109 value 1 | 0.332 | <2.23e-308 | 47475/44 |
| 20109 value 3 | 0.387 | <2.23e-308 | 47455/64 |
| 20112 value 10 | 0.858 | <2.23e-308 | 2114/125 |
| 20112 value 11 | 0.239 | <2.23e-308 | 2164/71 |
| 20112 value 12 | 0.976 | <2.23e-308 | 2159/75 |
| 20113 value 11 | 0.968 | <2.23e-308 | 2475/46 |
| 20113 value 3 | 0.161 | <2.23e-308 | 2410/126 |
| 20113 value 4 | 0.614 | <2.23e-308 | 2383/156 |
| 20113 value 5 | 0.707 | <2.23e-308 | 2274/272 |
| 20114 value 4 | 0.956 | <2.23e-308 | 1539/32 |
| 20114 value 5 | 0.409 | <2.23e-308 | 1508/65 |
| 41218 value 2 | 0.824 | <2.23e-308 | 644/123 |

Variables shown are those where P values of main analysis was >0.05 and P value of sensitivity analysis was < 2.36x10^-6^ (the Bonferroni corrected threshold).

^1^ In R the smallest possible number is 2.23e-308 (specified in .Machine$double.xmin).

Supplementary table I: Summary statistics comparing those who reported yes versus no for each nervousness phenotype

|  | **Nervous person (FID=1970)** | | | **Being a worrier (FID=1980)** | | | **Tense/highly strung (FID=1990)** | | | **Suffering from nerves (FID=2010)** | | |
| --- | --- | --- | --- | --- | --- | --- | --- | --- | --- | --- | --- | --- |
|  | Yes | No | OR (yes vs no) | Yes | No | OR (yes vs no) | Yes | No | OR (yes vs no) | Yes | No | OR (yes vs no) |
| **Continuous phenotypes (mean [SD])** | | | | | | | | | | | | |
| BMI (kg/m^2^) | 26.72 [4.76] | 27.60 [4.72] | 0.960  [0.958, 0.961] | 27.15 [4.80] | 27.69 [4.67] | 0.976  [0.975, 0.978] | 27.00 [4.91] | 27.46 [4.70] | 0.979  [0.977, 0.981] | 27.24 [4.85] | 27.43 [4.71] | 0.991  [0.989, 0.993] |
| Age at recruitment (years) | 56.62 [7.93] | 56.93 [8.01] | 0.995 [0.994, 0.996] | 56.77 [7.94] | 56.95 [8.06] | 0.997 [0.996, 0.998] | 56.25 [8.13] | 56.99 [7.95] | 0.989 [0.987, 0.990] | 55.91 [8.01] | 57.10 [7.97] | 0.982 [0.981, 0.983] |
| Townsend deprivation index | -1.39 [3.05] | -1.65 [2.88] | 1.030  [1.027, 1.033] | -1.56 [2.95] | -1.63 [2.89] | 1.008  [1.006, 1.011] | -1.26 [3.13] | -1.67 [2.86] | 1.048  [1.045, 1.051] | -1.32 [3.10] | -1.67 [2.87] | 1.040  [1.037, 1.043] |
| Age completed full time education (years) | 16.57 [2.15] | 16.70 [2.22] | 0.972 [0.967, 0976] | 16.63 [2.18] | 16.70 [2.24] | 0.986 [0.982, 0.990] | 16.52 [2.20] | 16.70 [2.20] | 0.962 [0.956, 0.967] | 16.59 [2.15] | 16.69 [2.21] | 0.978  [0.973, 0.983] |
| **Binary phenotypes (numbers [percentages])** | | | | | | | | | | | | |
| Sex: Female | 47,643 [61.67] | 127,337 [51.07] | 0.649  [0.638, 0.660] | 113,263 [61.07] | 62,677 [44.42] | 0.509  [0.502, 0.517] | 33,416 [59.96] | 141,089 [52.36] | 0.734  [0.721, 0.738] | 36,521 [52.86] | 136,642 [53.76] | 1.04  [1.02, 1.05] |
| Male | 29,618 [38.33] | 121,995 [48.93] |  | 72,194 [38.93] | 78,432 [55.58] |  | 22,317 [40.04] | 128,360 [47.64] |  | 32,564 [47.14] | 117,529 [46.24] |  |
| Depressed whole week ^1^: No | 8,550 [34.55] | 41,006 [50.37] | 1.923  [1.867, 1.981] | 23,202 [38.81] | 26,199 [56.45] | 2.044  [1.994, 2.095] | 5,373 [29.95] | 44,147 [50.21] | 2.359  [2.279, 2.442] | 7,327 [32.32] | 42,068 [50.93] | 2.17  [2.11, 2.24] |
| Yes | 16,198 [65.45] | 40,396 [49.63] |  | 36,586 [61.19] | 20,211 [43.55] |  | 12,567 [70.05] | 43,773 [49.79] |  | 15,344 [67.68] | 40,539 [49.07] |  |
| Smoking status:  Never | 43,687 [56.74] | 134,616 [54.17] | 0.901 [0.887, 0.916] | 102,477 [55.45] | 75,902 [53.96] | 0.942 [0.929, 0.955] | 28,901 [52.05] | 148,820 [55.41] | 1.145 [1.124, 1.166] | 37,057 [20.97] | 139,634 [55.12] | 1.053 [1.035, 1.071] |
| Ever | 33,310 [43.26] | 113,889 [45.83] |  | 82,343  [44.55] | 64,755  [46.04] |  | 26,628  [47.95] | 119,771  [44.59] |  | 31,780 | 113,716  [44.88] |  |

BMI: body mass index; SD: standard deviation; OR: odds ratio; FID: field identifier.

^1^ Answers to the question “Looking back over your life, have you ever had a time when you were feeling depressed or down for at least a whole week?”

Odds ratio for answering yes (comparison) versus no (reference group) to questionnaire item, for a one-unit increase in continuous variable (using variable units as described in column 1), or comparison group (indicated in column 1) versus baseline group for binary variables, or a one-category increase for ordinal categorical variables.

SUPPLEMENTARY FIGURES

Supplementary figure A: Venn diagram of UK Biobank subsamples use in our main, discovery and replication analyses

Discovery sample includes all participants included in our initial MR-pheWAS (2).

Replication sample includes all participants included in the main analysis on the full available (~350K) sample.

Initial discovery sample is not a subset of the main ~335K sample because when removing participants from each related pair different selections were made.

Supplementary figure B: Variable processing flow diagram showing logic from defined field type in UK Biobank data to test of association, with number of variables reaching each stage of processing flow

Variable processing flow diagram showing logic from defined field type specified by UK Biobank to test of association, and the number of variables at each stage of the processing flow. Triangular nodes at top of figure are field types defined by UK Biobank. Rectangular nodes show processing logic used to determine the data type assignment (oval), either continuous, ordered categorical, unordered categorical or binary, and hence finally, the type of test used: linear, ordinal logistic, multinomial logistic or logistic regression, respectively. Diamond nodes show points where variables may be removed.

Supplementary figure C: Comparison of main MR-pheWAS results compared to sensitivity analyses (additionally adjusting for assessment centre and genetic batch)

a) Comparison including all results

b) Same plot but restricting to results where main analysis P-value is between 10^-10^ and 10^-2^

Axis are on a log scale.

Main MR-pheWAS analysis: adjusted for age, sex and the first 10 genetic principal components.

Sensitivity analysis: adjusted for age, sex, first 10 genetic principal components, assessment centre and genetic batch.

Green dashed line: Bonferroni P value threshold; Blue dotted line: FDR P value threshold.

Details of the results that differ are given in Table H in S1 Text.

The points set at 10e-320 have values less than the smallest possible number in R (2.23e-308), specified in .Machine$double.xmin.

Supplementary figure D: Mr-Egger plots for follow-up analysis of anxiety traits

a) Nervous feelings (FID=1970) b) Being a worrier (FID=1980)

c) Tense / highly strung (FID=1990) d) Suffers from nerves (FID=2010)

FID: Field identifier.

Genetic association with BMI is the standard deviation change of BMI for a 1 dosage SNP increase. Genetic association with outcome is the increase in the log odds of outcome for a 1 dosage SNP increase.

Green dashed line: weighted median estimate. Blue solid line: MR-egger estimate.

Supplementary figure E: MR-Egger SIMEX plots

a) Nervous feelings (FID=1970) b) Being a worrier (FID=1980)

c) Tense / highly strung (FID=1990) d) Suffers from nerves (FID=2010)

FID: Field identifier.

Figure shows the causal estimates with MR-Egger (blue dot), causal estimate with increasing invalidity of NOME assumption (black dots), and causal estimate when NOME assumption holds (blue cross), estimated using extrapolation.

Supplementary figure F: Smoothed empirical distribution across SNP causal effects, of unweighted MBE

| a) FID=1970, phi=0.5   | b) FID=1970, phi=0.75   | c) FID=1970, phi=1   |
| --- | --- | --- |
| a) FID=1980, phi=0.5   | b) FID=1980, phi=0.75 | a) FID=1980, phi=1   |
| a) FID=1990, phi=0.5   | a) FID=1990, phi=0.75 | a) FID=1990, phi=1   |
| a) FID=2010, phi=0.5   | a) FID=2010, phi=0.75   | a) FID=2010, phi=1   |

FID: Field identifier.

x-axis: individual instrument ratio causal effect estimates.

FID=1970: nervous feelings; FID=1980: being a worrier; FID=1990: tense / highly strung; FID=2010: suffers from nerves.

Supplementary figure G: Smoothed empirical distribution across SNP causal effects, of weighted MBE

| b) FID=1970, phi=0.5   | a) FID=1970, phi=0.75   | c) FID=1970, phi=1   |
| --- | --- | --- |
| a) FID=1980, phi=0.5   | a) FID=1980, phi=0.75   | a) FID=1980, phi=1   |
| a) FID=1990, phi=0.5   | a) FID=1990, phi=0.75   | a) FID=1990, phi=1   |
| a) FID=2010, phi=0.5   | a) FID=2010, phi=0.75   | a) FID=2010, phi=1 |

FID: Field identifier.

FID=1970: nervous feelings; FID=1980: being a worrier; FID=1990: tense / highly strung; FID=2010: suffers from nerves.

x-axis: individual instrument ratio causal effect estimate.

**References**

1. Locke AE, Kahali B, Berndt SI, Justice AE, Pers TH, Day FR, et al. Genetic studies of body mass index yield new insights for obesity biology. Nature. 2015;518(7538):197–206.

2. Millard LAC, Davies NM, Gaunt TR, Davey Smith G, Tilling K. PHESANT: a tool for performing automated phenome scans in UK Biobank. Int J Epidemiol. 2017;dyx204.
